# Adaptive multi-stage domain unlearning for white-matter lesion segmentation

**DOI:** 10.3389/fmed.2026.1875760

**Published:** 2026-07-31

**Authors:** Domen Preložnik, Žiga Špiclin

**Affiliations:** Faculty of Electrical Engineering, University of Ljubljana, Ljubljana, Slovenia

**Keywords:** adaptive domain unlearning, comparative evaluation, domain generalization, image segmentation, open-source and reproducible, scanner variability

## Abstract

**Introduction:**

Inter-scanner variability in magnetic resonance imaging (MRI) adversely affects the diagnostic and prognostic quality of scans and necessitates the development of models that are robust to domain shift arising from the unseen scanner data. A review of recent advances in domain adaptation and domain generalization showed that the efficacy of strategies involving modifications or constraints on the latent space appears to be contingent upon the level and/or depth of supervision during model training.

**Methods:**

We propose an adaptive multi-stage domain unlearning (ADMU) technique to improve robustness to unseen scanner domains. Building on the state-of-the-art segmentation framework nnU-Net, we employ deep supervision at deep encoder stages by applying domain classifier unlearning, sequentially across these stages to reduce domain-discriminative latent features. Following the self-configurable approach of nnU-Net, the auxiliary feedback loop implements an adaptive backpropagation schedule for unlearning. Experiments were conducted on four public datasets (one for training, three for testing) to benchmark white-matter lesion segmentation methods. Five benchmark models and/or strategies, spanning passive to active domain-robust training strategies, were tested, and five state-of-the-art methods were compared.

**Results:**

AMDU demonstrated consistent, robust and improved cross-dataset segmentation performance on three test sets versus baseline nn-Unet variants. The advantage of AMDU was in enhanced lesion sensitivity with balanced false detections, resulting in good overall segmentation quality, as measured by segmentation overlap and relative lesion volume error. Compared to continuous domain unlearning, the adaptive scheduling balanced the adverse impact of unlearning onto the main segmentation task. Intensity-based preprocessing was found to be detrimental to segmentation performance.

**Discussion:**

The proposed AMDU strategy was shown to be complementary to data augmentation. Demonstrated for white-matter lesion segmentation it relied only the FLAIR modality, simplifying preprocessing to spatial normalization to brain atlas, with no intensity harmonization, for best cross-dataset segmentation performance. The source code is available at https://github.com/Pubec/nnunetv2-unlearning.

## 1 Introduction

**Figure 1:**
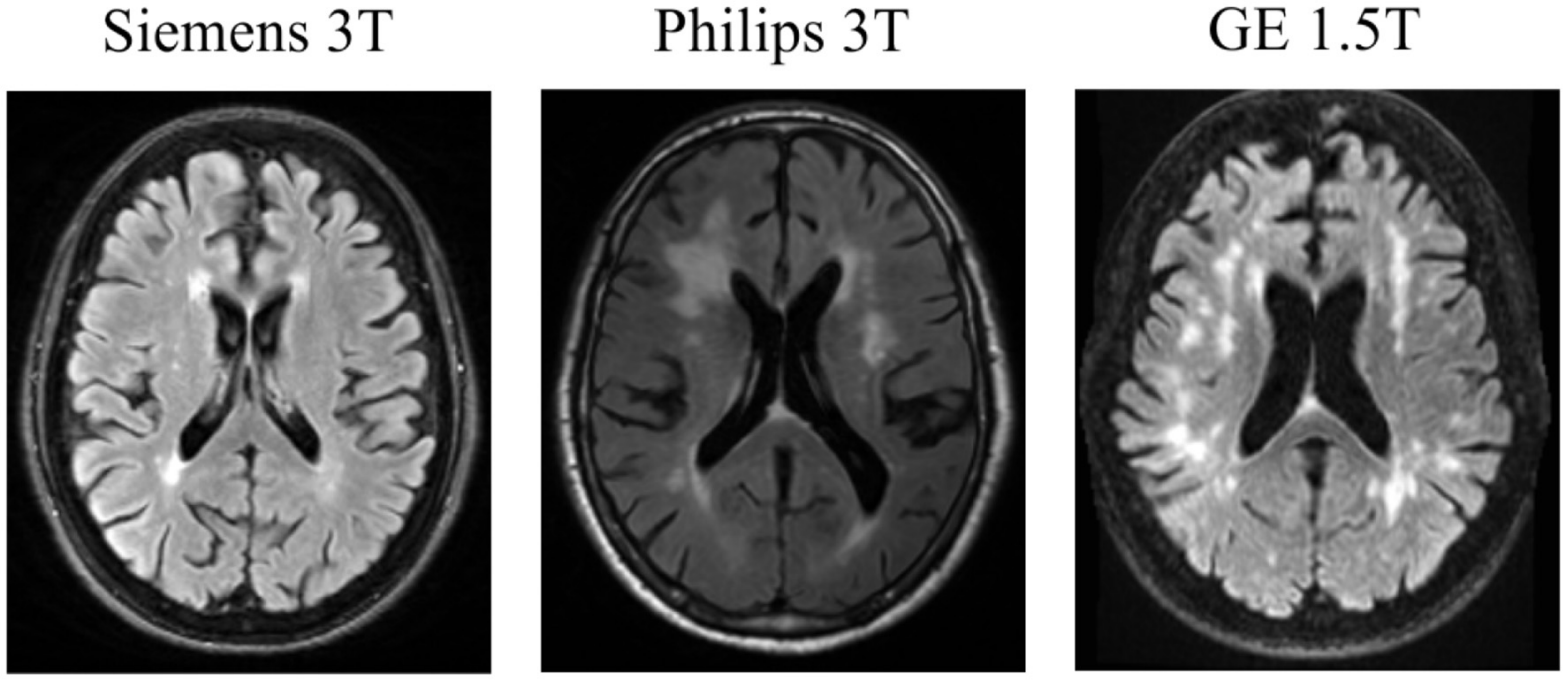
Comparison of MRI scans between different domains.

The proliferation of brain magnetic resonance imaging (MRI) in conjunction with deep learning (DL) image analysis enables state-of-the-art white-matter lesion (WML) segmentation (1–3). Segmentation should be accurate and robust, as the total number and volume of lesions quantified from MRI have been established as biomarkers for diagnosis, assessment of response to therapy (4), and future Multiple Sclerosis (MS) disease progression (5). However, inter-scanner variability in intensity contrast and noise distributions (Figure 1) remains the most detrimental factor for the diagnostic and prognostic quality of MRI scans (6). This variability undermines a segmentation model's ability to generalize to new domains (i.e., new scanners). Obtaining high-quality expert lesion annotations for new-domain data to fine-tune the model is often prohibitively time-consuming and impractical. These observations necessitate the development of broadly applicable segmentation models that are not restricted to the source domain on which they were trained.

The difficulty of consistently analyzing data from an unseen domain is increasingly highlighted in the DL literature. For example, Alzubaidi et al. (7) and the Google Research Team (8) refer to *underspecification* to explain the poor behavior of DL systems when deployed in real-world domains, regardless of the application area. When evaluating WML segmentation, the same phenomenon is apparent in the results of public WML segmentation challenges. In MICCAI 2016 MS Lesion Segmentation Challenge (MSSEG) results (9), the segmentation performances of all tested methods generalized well on the test set of the source scanner data, on which they were trained, but dropped substantially on the unseen test set (*Center 3*). Similar observations hold for the MICCAI 2021 MS New Lesions Segmentation Challenge (MSSEG-2) results (10). Even the top-ranking team's method exhibited a drop in DSC score from 0.52 (*seen*) to 0.26 (*unseen*). The domain shift, therefore, needs to be addressed accordingly.

We hypothesize that poor generalization of DL models across domains and domain overfitting mainly arise from an inability to discriminate between task- and domain-related features, from the enhancement rather than suppression of these features, and from other factors, such as inadequate data augmentation, MRI preprocessing and/or harmonization, and data imbalance. To address cross-domain robustness in WML segmentation, we propose adaptive multi-stage domain unlearning (AMDU) by augmenting nnU-Net with multi-stage encoder supervision through domain-classifier unlearning. The main methodological contributions of this study are:

(1) We propose an *adaptive multi-stage domain unlearning* strategy that reduces scanner-specific discriminability without requiring target-domain data during training;
(2) We introduce an adaptive learning–unlearning schedule that triggers unlearning according to domain-classifier behavior rather than a fixed alternation rule;
(3) We integrate the proposed strategy into nnU-Net (11), providing an open-source implementation for domain-affected segmentation tasks.

Empirically, the proposed AMDU improved cross-domain lesion segmentation performance on unseen datasets while maintaining competitive performance on seen data. We further found that intensity-based preprocessing was detrimental, whereas the proposed unlearning strategy complemented data augmentation and yielded competitive results compared with recent state-of-the-art methods, despite using only FLAIR input.

A note on terminology: we use *adaptive multi-stage domain unlearning* to denote the proposed mechanism, *adaptive learning–unlearning schedule* to denote its triggering strategy, and *source-only domain-robust training* to denote the experimental setting in which no target-domain data is used during training or adaptation. We therefore discuss the method in relation to the broader domain adaptation literature while evaluating it as zero-shot transfer to unseen domains.

## 2 Related work

Domain adaptation is a specific type of transfer learning in which domain features, i.e., a feature subspace defined by the source dataset, and task features, defined by labels and goals, are disentangled to enable knowledge transfer, e.g., by emphasizing task features and disregarding/suppressing domain-identifying features. This adaptation allows models to adequately apply prior knowledge to previously unseen target domain (12). In practice, cross-domain MRI segmentation methods span several distinct settings that should be differentiated clearly: (i) source-free or source-only domain generalization approaches, which aim to improve robustness without target-domain data during training; (ii) test-time or post-deployment adaptation approaches, which exploit target-domain information at inference or fine-tuning time; (iii) minimal-instance and few-shot adaptation approaches, which adapt using very limited target-domain samples; and (iv) multi-dataset minimal-instance adaptation studies, which assess such strategies across several external datasets.

Data and model diversification is a prospective strategy for source-only domain-robust training and is also commonly used within unsupervised domain adaptation (UDA) pipelines to aid model generalizability. Simple techniques involve aggressive data augmentation and the generation of synthetic data during training (13–16), provided that domain-identifying variations can be modeled reasonably well. Ensembling is an established approach for aggregating diverse model outputs, e.g., using multiview models (17, 18). In LST-AI (19), which is based on an ensemble of three 3D U-Nets (20, 21), each trained with deep supervision at one or two layers and various combinations of losses to promote model diversity, showed good generalization performance over state-of-the-art models on unseen datasets. A related, but more explicitly domain-generalization-oriented, direction is represented by DeepLesionBrain (22), which simulates acquisition variability and MRI artifacts to improve robustness across datasets without target-domain adaptation during training.

Data harmonization techniques for domain-shift mitigation aim to reduce variability arising from differences in acquisition protocols, scanners, or sites. A common prospective form is MRI preprocessing (23). It may include registration to standard brain atlas space, non-brain tissue removal, bias field correction, denoising, and intensity normalization. The preprocessing is standardized in many public segmentation challenges, which usually provide preprocessed scans alongside raw ones; e.g., Commowick et al., (10), Kuijf et al. (24), Commowick et al., (25). The downside is that bias correction or denoising may inadvertently remove task-relevant features, potentially degrading the downstream segmentation model's effectiveness.

As an alternative to centralized harmonization, distributed learning paradigms such as federated learning have recently emerged, enabling multi-site model training without sharing raw patient data while still addressing inter-site heterogeneity; for example, Hindawi et al. (26) demonstrated the feasibility of federated nnU-Net training for multiple sclerosis lesion segmentation across geographically distributed clinical centers. However, compared with federated learning, UDA can offer a more practical and often more effective solution for post-deployment domain shift, as it explicitly optimizes model adaptation to a previously unseen target domain using only unlabeled target-domain data, without requiring access to participating institutions, repeated collaborative training, or re-exposure to the original distributed datasets. This broader target-adaptive family includes both test-time adaptation and minimal-instance adaptation, in which some target-domain information is made available after deployment rather than being suppressed already during source training.

Voxel-level harmonization methods such as Ravel (27), ComBat and its extensions (28–30) have been successfully used to evaluate and correct harmonization effects in diffusion MRI and longitudinal structural imaging, but they are applied retrospectively. Image-level harmonization, such as MURD (31), seeks to translate scanner-specific appearance while preserving anatomy, but it risks altering or hallucinating anatomically relevant details, which is particularly problematic for pathology-sensitive tasks like WML segmentation. In general, these techniques require recalibration each time new scanners or acquisition sites are added to the dataset and rely on demographic subject information, which is not always available.

Feature-level methods, such as MSCDA (32), align representations using contrastive objectives but depend on carefully designed positive/negative pairs and often assume access to all domains at inference, limiting their clinical applicability. Domain-randomized synthetic training, exemplified by SynthSeg (33), offers scalability yet may fail to capture realistic pathological variability because it relies on fully synthetic images.

Generative methods have also been proposed to address domain shift in MRI. ImUnity (16) is a self-supervised VAE-GAN-based harmonization approach that removes scanner-specific biases and preserves clinical features, but is currently limited to T1-weighted scans and may introduce task-irrelevant features due to the inherent choice of the target domain in the decoder. SAMSEG (34) uses a forward probabilistic model with latent variables to constrain WML shape and location for automated segmentation. A more recent target-adaptive study has also explored few-shot adaptation across datasets. In particular, Coppola et al. (35) studied cross-domain few-shot adaptation in a multi-modal setting, training on one dataset and adapting the evaluation to external public datasets, thereby representing both a minimal-instance and a multi-dataset adaptation strategy. However, generative, harmonization, and target-adaptive methods share an important practical trade-off: they typically rely on additional modalities, target-domain samples, or post-deployment adaptation steps that are not available in strict source-only deployments. They also share two broader limitations: (i) reduced robustness in cases with marked biological or pathological variability (e.g., severe atrophy or extensive WMLs), and (ii) they may not consistently improve downstream segmentation performance.

Recent implicit, representation-free techniques like domain unlearning involve training a domain classification network based on encoder and/or decoder outputs of the main task network, and unlearning it via a domain classifier confusion loss to nullify encoding of domain-related information (36, 37). This is analogous to the confusion module at the bottleneck layer in ImUnity (16), a self-supervised Variational AutoEncoder developed for T1-weighted MRI scan normalization. Another approach fuses latent space representations with respect to domain-identifying features via latent embeddings, while contrasting them for the main task (38). Similarly, a disentanglement of anatomical and domain feature representations was achieved by penalizing interdependence among feature maps via mutual information minimization (39). Based on these advances, we hypothesize that the efficacy of strategies involving modifications or constraints on the latent space depends on the level and/or depth of model supervision during training.

Taken together, the existing literature shows that domain-shift mitigation in WML segmentation can be pursued through source-free robustness induction, test-time or few-shot target adaptation, multi-dataset minimal-instance adaptation, harmonization, and representation-level suppression. The identified limitations of these strategies motivate our focus on representation-level unlearning, which avoids assumptions about generative translation, does not require target-domain samples at inference, and remains compatible with heterogeneous multi-scanner FLAIR datasets in real-world scenarios.

Efficacy of the segmentation advances should be determined through a comparative analysis with state-of-the-art segmentation frameworks such as nnU-Net (11), Transformer networks (40), attention-guided networks (41), or their ensembles (42), which is not always the case. As pointed out in Isensee et al. (43) and Christodoulou et al. (44), the use of inadequate baselines may leave a gap in understanding the relative effectiveness of novel approaches. While based on a simple U-Net architecture and using aggressive data augmentation, the nnU-Net proved surprisingly effective as a source-only robust transfer baseline. We aim to address the same goal, i.e., with model training performed on *seen* data, while testing on *unseen* domains, in the context of WML segmentation in MRI scans of different scanner vendors and sites.

## 3 Methods

### 3.1 nnU-Net baseline

**Figure 2:**
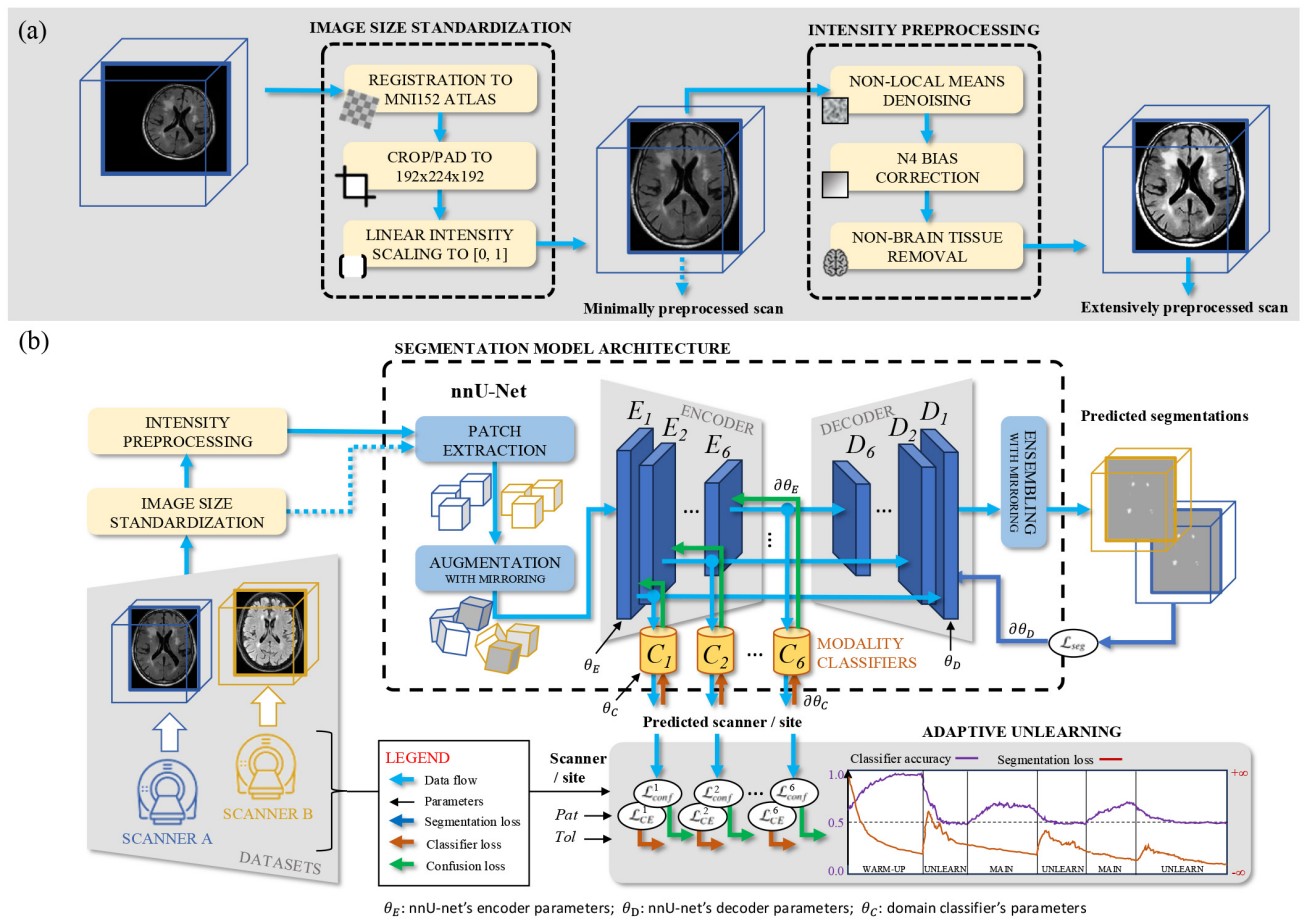
**(a)** MRI preprocessing and **(b)** adaptive multi-stage domain unlearning with nnU-Net.

Our baseline model was the default nnU-Net self-configuring framework.^1^ The *3D-fullres* model is depicted in Figure 2, with the encoder consisting of six stages (denoted with *E1–6*), each consisting of two consecutive Conv blocks of 3 × 3 × 3 convolutional layers, instance normalization (IN), and leaky rectified linear unit (lReLU) activation. In the top stage, the first block had a stride of 1 in the convolutional layer, while the respective stride was 2 in the other stages to achieve downsampling. The decoder consisted of six stages *D1–6*, with skip connections between the respective encoder blocks *E1–6* at the same resolution. Each decoder block consisted of a 2 × 2 × 2 transpose convolution with stride 2, IN, and an lReLU activation, followed by a Conv block. For training segmentation models, a Dice Similarity Coefficient (DSC) based loss (45) is a common (non) overlap measure defined as:


$$\begin{array}{l} L_{\text{DSC}}=1-\frac{2\times \text{TP}}{2\times \text{TP}+\text{FP}+\text{FN}}, \end{array}$$ (1)

where TP, FP, and FN denote the true positive, false positive, and false negative voxel labels, respectively. Similarly, the Cross Entropy (CE) loss (46) measures pixel-wise classification performance:


$$\begin{array}{l} L_{CE}=-\overset{N}{\underset{n=1}{\sum }}t_{n}\log y_{n}, \end{array}$$ (2)

where *N* is the number of classes (i.e., pixel labels for the segmentation task), *t*_*n*_ is the true probability, and *y*_*n*_ is the predicted probability for class *n*. The nnU-Net employs the sum of DSC and CE as the segmentation loss function:


$$\begin{array}{l} L_{seg}=L_{\text{DSC}}+L_{\text{CE}}. \end{array}$$ (3)

The default nnU-Net trainer uses patching into 128 × 128 × 128 chunks and implements a comprehensive data augmentation consisting of a sequence of input/output image/label manipulation steps. Each step is applied with random parameters according to a predefined probability in the following order: (i) spatial transformation by image/label rotation and scaling, (ii) addition of Gaussian noise, (iii) Gaussian blurring, (iv) brightness, (v) contrast and (vi) gamma intensity manipulation on the input image, (vii) low resolution (thick slice) simulation of the input image, and (viii) input/output patch mirroring applied symmetrically to the input/output image/label patches.

During inference, the nnU-Net ensembles predicted segmentation probability maps for original and mirrored image patches by averaging. The comprehensive augmentation strategy and ensembling render nnU-Net a robust, state-of-the-art deep learning model for segmentation tasks. Posterior lesion probability maps were binarized at the default nnU-Net threshold of 0.5 to obtain final segmentation masks used in all subsequent voxel-wise, volumetric, and lesion-wise evaluations.

### 3.2 nnU-Net with multi-stage unlearning

To enable multi-stage unlearning, the output of each encoder stage (*E1–6*) was fed to the corresponding domain classifier (blocks *C1–6*). Each classifier *Cx* consisted of *x*−1 blocks of 3 × 3 × 3 convolution layers with stride 2, IN and lReLU, to achieve a feature map size of 4 × 4 × 4. This was superseded by two Conv blocks, a flatten layer, and a fully connected layer with *N*_*d*_ outputs corresponding to the number of *seen* domains. Each classifier was trained by minimizing categorical CE loss as in Equation 2, with *N* equal to *N*_*d*_, which is the number of domains seen during training.

The final model included one *C* block for each *E* stage (six pairs in total), with the *C* blocks trained specifically for features output by the corresponding *E* stage. For domain unlearning purposes we computed *confusion loss* based on output of each *C* block as the Kullback–Leibler divergence:


$$\begin{array}{l} L_{conf}=-\overset{N}{\underset{i=1}{\sum }}p_{i}\log\left(\frac{p_{i}}{q_{i}}\right) \end{array}$$ (4)

where *p*_*i*_∈*P* are the domain classifier's posterior probabilities and *q*_*i*_∈*Q* are the target uniform probabilities of domain classes. Ideally, *q*_*i*_ = 1/*N*_*d*_, such that noninformative predictions (e.g., *p*_0_/*p*_1_ = 0.5/0.5 in case of two domains) yield a minimal, while highly informative predictions (e.g. *p*_0_/*p*_1_ = 1.0/0.0 in case of two domains) a maximal value of *confusion loss*. In this way, an informative classifier output that identifies the source image domain beyond random choice motivates task/domain feature disentanglement and reduces domain-discriminative features. Namely, we backpropagate $L_{conf}$ through the corresponding and preceding encoders, encouraging them to reduce domain-discriminative features. As the classifier predictions become less informative, we interpret this as reduced domain discriminability available to the classifier, rather than as definitive proof that all domain information was removed by the encoder. This *unlearning* process has to balance with the main segmentation *learning* task, for effective task/domain feature disentanglement.

### 3.3 Learning–unlearning schedule

The learning/unlearning schedule is detailed in Algorithm 1. First, during the *warm-up stage* (denoted by the number of epochs *e*_*w*_), the main task training takes place, in which: (i) the model is updated for the main segmentation task by minimizing the $L_{seg}$ (Equation 3) and (ii) all the classifiers *C1–6* are trained for domain classification task. The features output by each encoder block are passed to corresponding classifier, which is trained using the $L_{\text{CE}}$ (Equation 2).

Algorithm 1 Adaptive multi-stage domain unlearning. 
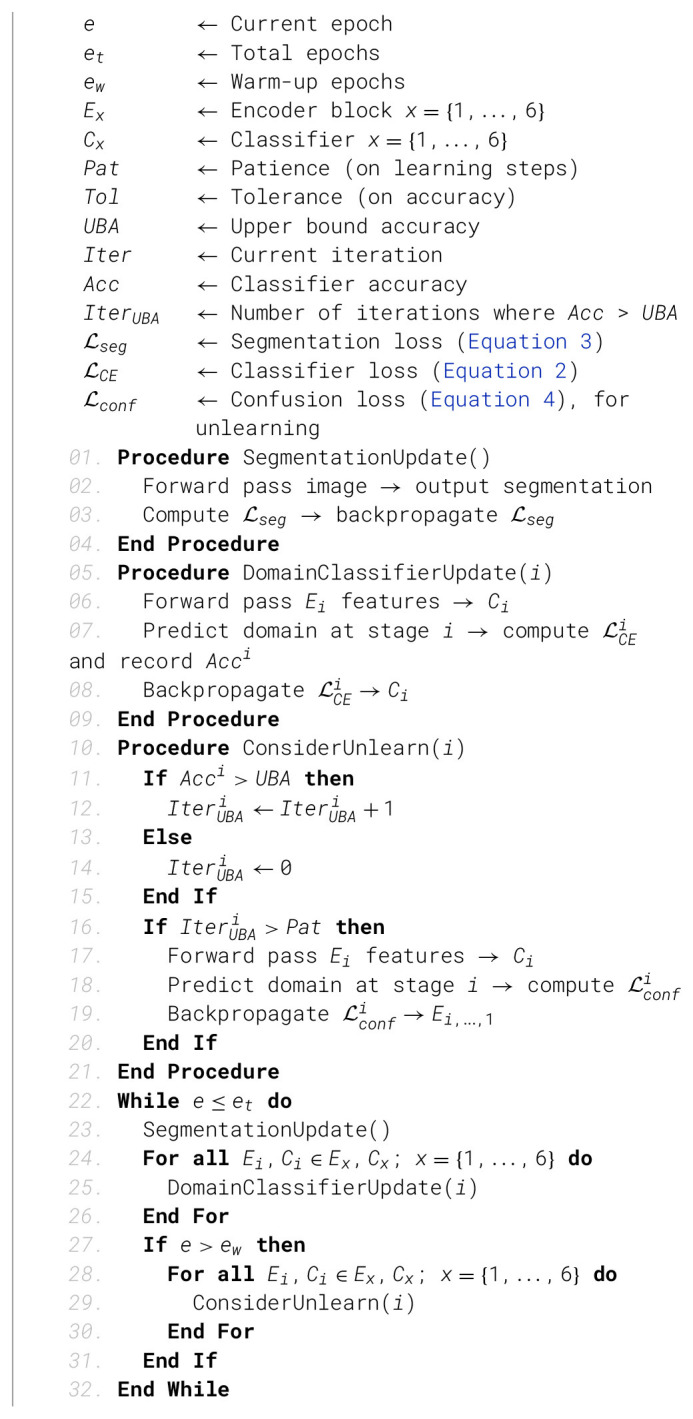

In the subsequent *learning-unlearning stage* steps (i) and (ii) were followed by (iii) unlearning of encoder blocks by backpropagation of $L_{conf}$, steming from domain classifier predictions. Before computing each $L_{conf}$ for *C1–6*, a new forward pass had to be performed, since backpropagating $L_{conf}$ causes change of encoder block's forward features. For each classifier *C1–6*, the corresponding $L_{conf}$ was computed based on preceding encoder block features, hence, the $L_{conf}$ was backpropagated via all the preceding encoder blocks. As such, the domain classifier at the U-net bottleneck, will update all *E1–6* blocks of the encoder.

In each iteration, we form a batch of the same number of images from each source domain. For instance, a batch of eight input images from two source domains included four images from each domain. Providing an unequal number of images per domain impedes model unlearning ability, since the confusion loss function (Equation 4) optimizes the model toward non-informative predictions, which are best discernible at equal source domain distributions.

#### 3.3.1 Adaptive schedule

Following the self-configuring philosophy of nnU-Net, we employ an adaptive *learning–unlearning* schedule. It is grounded in prior domain-adversarial and domain-confusion study (47, 48), and in information-bottleneck theory, which suggests that task-agnostic, MRI-scanner-specific nuisance signals reside in higher-level representations (49), thereby rendering unlearning effective. However, in a prior study, MRI unlearning from the bottleneck layer (36) was rather ineffective, despite hyperparameter tuning. This necessitates a curriculum-style patience mechanism (50) that triggers training changes only when a stable above-chance domain signal is detected.

Concretely, for a domain classifier *C*_*i*_ operating at a given encoder depth we compute an *upper-bound accuracy* (UBA) as:


$$\begin{array}{l} \text{UBA}=\frac{1}{N_{d}}+Tol,\;Tol\in \left[0,1-\frac{1}{N_{d}}\right], \end{array}$$ (5)

where *N*_*d*_ is the number of domains (e.g., scanners) and *Tol* is a small tolerance above random chance (0.5) that indicates a practically meaningful level of domain discriminability. While monitoring accuracy $Acc_{t}^{i}$ of classifier *C*_*i*_ at iteration *t* a counter $Iter_{UBA}^{i}$ of consecutive iterations where $Acc_{t}^{i}>\text{UBA}$ is maintained. When $Iter_{UBA}^{i}$ reaches the patience threshold *Pat*, the training alternates to an unlearning (gradient reversal) step for the particular classifier. Specifically, the confusion loss $L_{\text{conf}}^{i}$ is backpropagated via corresponding and previous encoders (*E*_*j*_; *j* = 1, …, *i*) in order to force the classifier toward random chance accuracy. When in effect, unlearning is applied alternately with the segmentation loss $L_{seg}^{i}$ in to preserve segmentation performance.

## 4 Experiments

### 4.1 Datasets

Four public MRI datasets containing cases with WMLs were employed in this study, namely the White-Matter Hyperintensities (WMH) 2017 Segmentation Challenge (24), the MSSEG (25), MS Ljubljana dataset (MSLJ) (51) and ISBI 2015 training dataset (52). Details about datasets are summarized in Table 1, with reported train/test data splits as used later in experimental evaluation.

**Table 1:** Dataset information and train/test splits as used in experimental evaluation.

Source/subset	Vendor	FS	AM	Train	Test
WMH/seen	Philips	3T	2D	20	30
	Siemens	3T	2D	20	30
WMH/unseen	Philips	3T	3D		10
	GE	3T	3D		30
	GE	1.5T	3D		10
MSSEG/unseen	Philips	3T	3D		15
	Siemens	3T	3D		15
	Siemens	1.5T	3D		14^†^
	GE	3T	3D		8
MSLJ/unseen	Siemens	3T	3D		30
ISBI/unseen	Philips	3T	2D		21

FS, field strength; AM, acquisition mode. ^†^Originally 15, but one case (msseg-test-center07-08) was removed due to incorrect reference mask.

### 4.2 Preprocessing

All FLAIR scans were preprocessed from raw acquisitions in the same manner: (i) linearly registered to the T1-weighted MNI 2009c brain atlas space (53), (ii) intensity range linearly rescaled to the [0–1 interval, and (iii) cropped to size 192 × 224 × 192, containing the whole brain. Such a preprocessing pipeline standardized the scan dimensions and intensity range, but intentionally did not modify the tissue contrasts, bias field, and noise distributions. To assess the impact of intensity preprocessing (itself a domain-shift mitigation strategy) on cross-domain performance of the WML segmentation models, we additionally evaluated an extended preprocessing pipeline that included the aforementioned three steps and subsequently applied: (iv) N4 bias correction (54), (v) adaptive non-local means denoising (55), and (vi) non-brain tissue removal. Computations were performed using the AntsPy package^2^ (56).

### 4.3 Experimental setup

The proposed model and four benchmark strategies were applied to the WMH seen-train subset (Table 1), using 32 cases for training and eight for validation and hyperparameter tuning. The four benchmarks were: (i) the nnU-Net without data augmentation; (ii) the default nnU-Net; (iii) the default nnU-Net applied to N4 bias-corrected and denoised train and test subsets; and (iv) a Dinsdale et al. (36) unlearning approach implemented into nnU-Net. Weight initialization and the order of the training data were randomized using a fixed seed. Three experiments were performed, as detailed in the following three paragraphs.

Ablation study involved the evaluation of the proposed approach on WMH's unseen subset of 50 scans from three scanners. We systematically explored unlearning strategies and schedules, evaluated their impact on the WML segmentation performance and generalization capability, and determined the best model. To avoid bias, these scans were not used further for evaluation.

Impact of preprocessing, augmentation and unlearning on segmentation and generalization performance was analyzed by comparing the best proposed model as determined in the ablation study against the default nnU-Net (with and without data augmentation), and the default nnU-Net applied to bias-corrected and denoised data. To further analyze the impact of unlearning, we compared the best proposed model against nnU-Net with unlearning implemented as in Dinsdale et al. (36), which uses a single-domain classifier at the bottleneck layer and a fixed learning-unlearning schedule. Quantitative evaluation was performed on three unseen test datasets (MSSEG, MSLJ, and ISBI) on a total of 103 FLAIR scans. Additionally, evaluation was performed on 60 test FLAIR scans from the *seen* WMH dataset (different subjects but the same scanners as in training) to assess the impact of scanner variability.

State-of-the-art comparison was then conducted using published results from methods evaluated on the same public unseen test sets, enabling contextual comparison using shared benchmarking data rather than retraining all external methods within our experimental pipeline. In addition to source-only and multimodal baselines, this comparison now explicitly includes test-time adaptation operating points with progressively increasing target-domain supervision, namely nnUNet fine-tuning with 1, 3, or 5 target-domain cases from Coppola et al. (35) on MSSEG and ISBI, as well as the one-shot adaptation model of (57) on ISBI. This design enables a more direct comparison between our source-only train-time adaptation strategy and post-deployment target-adaptive methods under low-resource supervision budgets.

Results are reported in corresponding subsections of Section 5.

### 4.4 Evaluation metrics

Output segmentation maps were evaluated using established metrics, such as the DSC ($1-L_{\text{DSC}}$, cf. Equation 1) and the TP Rate (TPR) defined as:


$$\begin{array}{c} \text{TPR}=\frac{\text{TP}}{\text{TP}+\text{FN}}. \end{array}$$ (6)

We further employed lesion-specific metrics, such as lesion-wise TPR (LTPR) (Equation 7) and lesion-wise False Discovery Rate (LFDR) (Equation 8), computed on the binarized segmentation masks using a predefined *intersection-over-union threshold* at *t*_*IOU*_ = 0.05. Specifically, the LTPR was defined as the ratio between the number of predicted lesions that overlap with lesions in the ground-truth, with percentage overlap higher than *t*_*IOU*_, and the total number of lesions in the ground-truth, namely:


$$\begin{array}{c} \text{LTPR}=\frac{\text{\#(LTP)}}{\text{\#(RL)}}, \end{array}$$ (7)

where LTP stands for *Lesion True Positives* and RL for *Reference Lesions*, and operator #(·) denotes *lesion count*, obtained via 18-connected neighborhood labeling operator.

The LFDR was defined as the ratio between the number of predicted lesions that do not overlap with any lesions in the ground-truth (overlap less than *t*_*IOU*_) and the total number of predicted lesions, and computed as:


$$\begin{array}{c} \text{LFDR}=\frac{\text{\#(LFP)}}{\text{\#(PL)}}, \end{array}$$ (8)

where LFP stands for *Lesion False Positives* and PL for *Predicted Lesions*.

Volumetric discrepancy between predicted and ground-truth segmentations was evaluated by the Relative Volume Error (RVE), defined as follows:


$$\begin{array}{c} \text{RVE}=\frac{\left|V\left(PL\right)-V\left(RL\right)\right|}{V\left(RL\right)}, \end{array}$$ (9)

where *V*(*L*) represents the volume obtained via pixel count of the specified label *L*.

To compare the overall performance in a set of different methods (or models or strategies), accounting for all aforementioned metrics, we used an average rank score defined as:


$$RS=\frac{1}{5}\cdot \left[R(DSC)+R(TPR)+R(LTPR)+R(LFDR)+R(RVE)\right]$$ (10)

where *R*(*m*) is the ranking of the method in the set according to a particular metric *m*. When having *M* methods in comparison, RS is in the range [1.0, *M*].

To analyze the effectiveness of unlearning, we also compute for each domain classifier *C*_*i*_ its accuracy as *Acc*^*i*^ = *TP*+*TN*/(*TP*+*TN*+*FP*+*FN*).

In the results, we report the mean (μ) and standard deviation (SD), or, where indicated, the bootstrap 95% confidence interval (CI) for all evaluated metrics. In performance comparisons, we used the Wilcoxon signed-rank test (58) or, where applicable, the independent-samples *t*-test to compare metric values of our proposed strategy against those of the other tested models or strategies. To adjust for multiple pairwise comparisons, we used Bonferroni correction by multiplying the obtained *p-values* by the number of methods in comparison. If the *adjusted p-value* was less than the significance threshold α = 0.05, we considered the observed difference statistically significant.

## 5 Results

### 5.1 Ablation study

Results in Table 2 provide a comparison of the effectiveness of unlearning strategies, exploring the hyperparameter space by varying: (i) stages that are unlearned (indicated by checkmarks and corresponding to stages as depicted in Figure 2); (ii) the ratio of learning to unlearning training steps (LUR); for instance, setting LUR to 5–1 indicates a schedule of five learning iterations of the main task and one iteration of encoder unlearning; (iii) the parameters of the adaptive unlearning schedule, i.e., patience (Pat) and tolerance (Tol) (Equation 5) for the best adaptive model. In case of using the *adaptive* learning-unlearning strategy as described in Section 3.2, we simply indicated specific stages that *were* being unlearned, but for which the proposed strategy controlled the execution of unlearning steps as described in Section 3.3.

**Table 2:** Ablation study results on the 2017 WMH segmentation challenge's unseen test split.

Unlearning stages						LUR	Unseen data - *not used for model training: Philips 3T 3D, GE 3T 3D, GE 1.5T 3D*					
1	2	3	4	5	6		DSC↑	TPR↑	LTPR↑	LFDR↓	RVE↓	RS↓
					✓	1–1	**0.719** **±0.100**	0.794 ± 0.116	0.717 ± 0.097	**0.251** **±0.113**	**0.262** **±0.235**	3.0
					✓	Adaptive	0.712 ± 0.099	0.805 ± 0.115	0.759 ± 0.103	0.294 ± 0.136	0.308 ± 0.272	3.2
			✓	✓	✓	5–1	0.674 ± 0.120	0.832 ± 0.101	**0.813** ± 0.095	0.474 ± 0.185	0.527 ± 0.449	3.4
			✓	✓	✓	Adaptive	0.715 ± 0.105	0.814 ± 0.120	0.764 ± 0.095	0.313 ± 0.136	0.316 ± 0.250	**2.8**
✓	✓	✓	✓	✓	✓	3–1	0.642 ± 0.132	**0.856** **±0.096**	0.741 ± 0.104	0.347 ± 0.185	0.763 ± 0.589	4.2
✓	✓	✓	✓	✓	✓	Adaptive	0.629 ± 0.122	0.813 ± 0.114	0.792 ± 0.110	0.453 ± 0.172	0.687 ± 0.597	4.4

Values are mean ± standard deviation, while bold indicates the best result for a particular evaluation metric. LUR is the ratio of the number of learning steps to unlearning steps.

**Figure 3:**
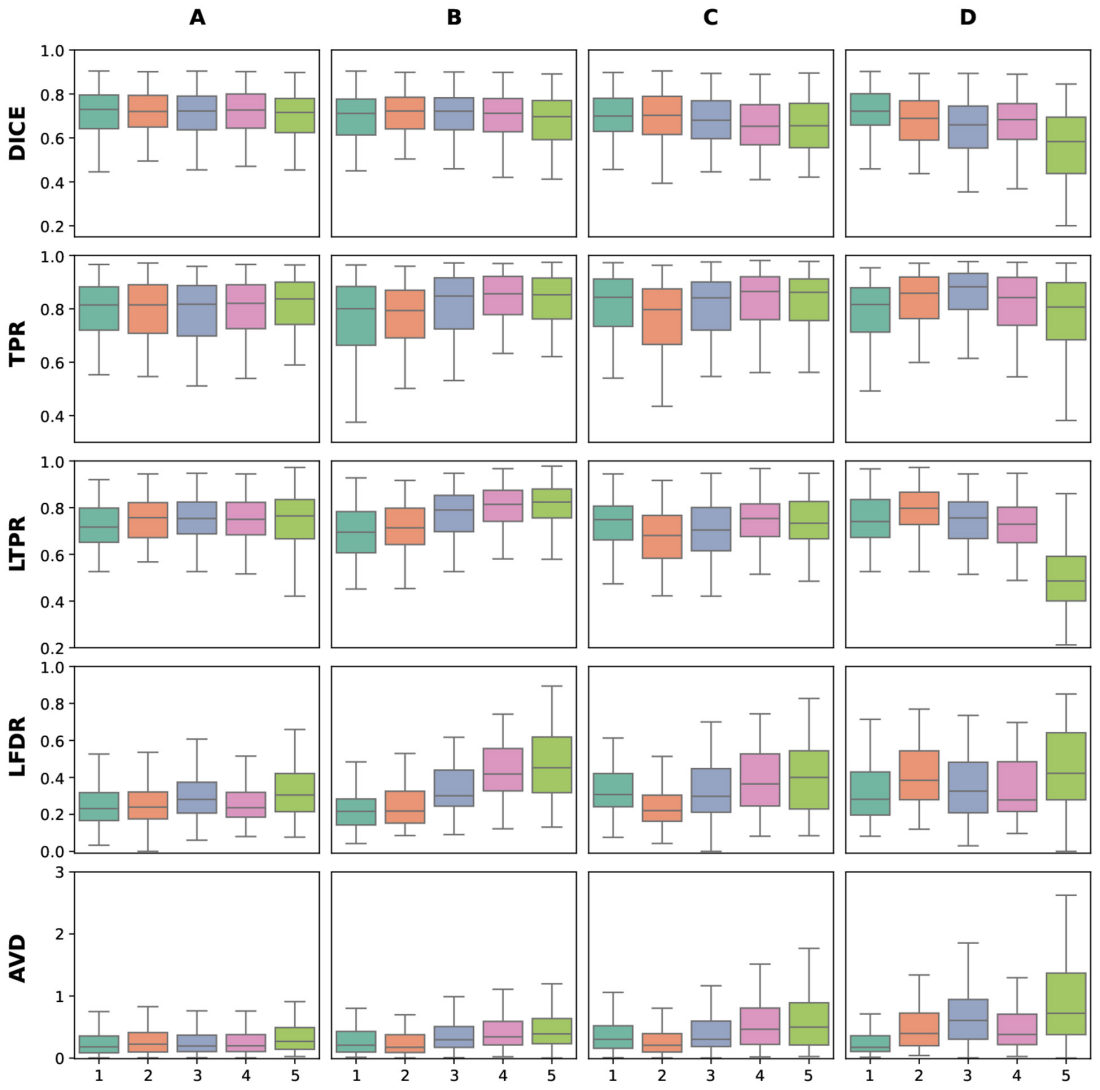
Ablation study metric values with respect to learn-to-unlearn ratio (LUR = 1–1, 2–1, 3–1, 4–1, 5–1), for various unlearning stages: **(A)** from bottleneck layer 3, **(B)** layers 4, 5, and 6, **(C)** layers 1, 2, and 3, **(D)** all layers 1–6.

We explored five manual LUR settings (1–1, 2–1, 3–1, 4–1, 5–1) across four representative stage configurations: bottleneck-only unlearning; stages 1–3; stages 4–6; and stages 1–6. Table 2 reports, for these explored configurations, the settings that achieved the best value for each evaluation metric, whereas Figure 3 visualizes the broader behavior across the manual LUR settings. Establishing a single optimal set of unlearning stages and LUR is challenging, since different segmentation metrics favor different settings (as indicated by the bolded values in Table 2). Although the adaptive model did not achieve the best value in any individual metric, it provided the most balanced overall performance across metrics. The average ranking score RS was lowest for the adaptive model with deeper unlearning stages (layers 4, 5 and 6), at 2.8 (Equation 10). We therefore selected this configuration for subsequent experiments and refer to it as the AMDU model.

Results in Figure 3 show that (i) most stage settings exhibited better performance with balanced LUR of 1–1, while LUR of 5–1 proved to be the worst across most metrics and stages. An exception is TPR and LTPR for setting *B*, which has a very high LFDR of 0.527 (Equation 6 and 7). (ii) We notice overall poor performance in setting *D*, with lowest values for all metrics at LUR 5–1. As expected (iii), with the increase in the LUR ratio, the TPR and LTPR tend to increase (cf. settings *A, B*, and *C*), since performing more learning vs. unlearning steps benefits the main segmentation task. However, (iv) performance varies more in settings *C* and *D*, whereas *A* and *B* yield consistent results across all LURs.

Table 3 shows that the nnU-Net and AMDU post-warm-up stages were able to identify the domain with nearly perfect accuracy across all six encoder stages. After unlearning the upper three stages, the accuracy of the corresponding domain classifiers remained in the range 77%–85%, indicating a limited reduction in domain discriminability. When unlearning the deeper stages (4, 5, and 6), the corresponding classifier accuracies were 48%–67% and 48%–50% for stages 5 and 6, respectively. Interestingly, when unlearning all stages, the reduction in domain discriminability was generally less effective, leading to near-chance accuracy only at the bottleneck (*i*=1–5: 99–65%; *i* = 6: 52%). Within the explored configurations, these findings indicate that unlearning deeper encoder stages was associated with the strongest reduction in scanner-related signal available to the auxiliary classifiers, alongside the most balanced segmentation performance.

**Table 3:** Domain classifier accuracy, post warm-up and post-unlearning, per stage of the nnU-Net AMDU on the 2017 WMH dataset's seen train split, comparing first- and last-three and unlearning of all stages.

Model		Classifier accuracy, *Acc^i^*					
Unlearning stages	Step	i = 1	i = 2	i = 3	i = 4	i = 5	i = 6
nnU-Net*		100%	100%	100%	100%	100%	100%
nnU-Net* AMDU	Post warm-up	100%	100%	100%	100%	100%	99%
Unlearning stages: 1 –3	Post unlearning	**85%**	**77%**	**80%**	98%	99%	91%
nnU-Net* AMDU	Post warm-up	100%	99%	99%	99%	99%	99%
Unlearning stages: 4–6	Post unlearning	100%	100%	100%	**67%**	**48%**	**50%**
nnU-Net* AMDU	Post warm-up	100%	99%	99%	99%	99%	99%
Unlearning stages: 1–6 (all)	Post unlearning	**99%**	**90%**	**76%**	**65%**	**67%**	**52%**

Values in bold indicate stages actively unlearned. *Predefined default trainer in nnU-Net.

**Figure 4:**
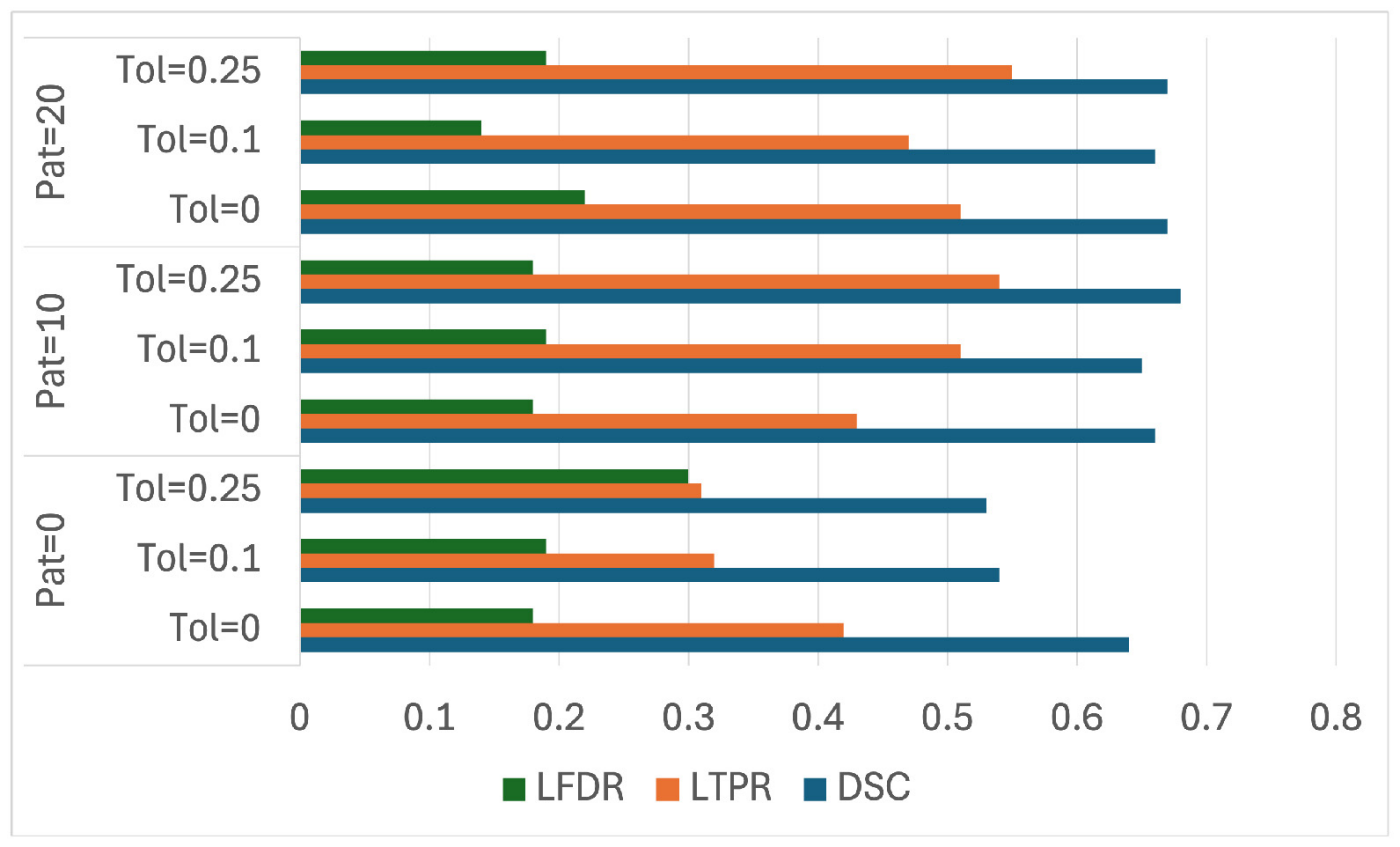
Impact of adaptive unlearning schedule parameters patience (Pat) and tolerance (Tol) on the lesion segmentation performance [model with last three unlearning stages (4, 5, and 6)].

Figure 4 shows the impact of adaptive unlearning schedule hyperparameters, i.e., patience (Pat) and tolerance (Tol), on the segmentation performance of the proposed AMDU model. Continuous unlearning (*Pat* = 0) yielded the poorest overall performance, indicating that immediate suppression of domain-related features does not allow sufficient learning of the main segmentation task. Increasing patience improved overall performance by allowing longer task-focused optimization before unlearning was triggered. The tolerance parameter *Tol* determines how far above chance the domain classifier accuracy must rise before unlearning is applied; a higher *Tol* therefore enforces a stricter criterion for activating feature suppression. Overall, the best performance was obtained with *Pat* = 20 and *Tol* = 0.25, which were used in subsequent experiments.

### 5.2 Impact of preprocessing, augmentation, and unlearning

Lesion segmentation results for the proposed and other models and domain-robustness strategies are shown in Table 4. The WMH results on the seen subset indicate that values of DSC and RVE for the proposed AMDU model are on par with the default nnU-Net. On the other hand, the proposed model comparatively improved TPR and LTPR, while LFDR increased (all noted differences were statistically significant). Despite a larger LFDR, indicating more incorrectly detected lesions. However, these were small FP lesions, as indicated by the lower RVE value in the proposed model.

**Table 4:** Segmentation evaluation on seen (WMH) and three unseen (MSSEG, MSLJ, ISBI) datasets, using respective seen and unseen test splits (Table 1).

Model	DSC↑	TPR↑	LTPR↑	LFDR↓	RVE↓	RS↓
WMH-*seen test dataset*						
nnU-Net no DA	0.750 ± 0.120^†^	0.775 ± 0.132^†^	0.680 ± 0.160^†^	0.390 ± 0.160^†^	0.220 ± 0.353	3.2
nnU-Net^*^	**0.773** **±0.108**	0.785 ± 0.129^†^	0.679 ± 0.103^†^	**0.210** **±0.131**	0.205 ± 0.388	2.0
nnU-Net^*^ preprocessing^**^	0.732 ± 0.123^†^	0.748 ± 0.149^†^	0.630 ± 0.105^†^	0.219 ± 0.137^†^	0.232 ± 0.371	3.8
Dinsdale et al. (36)	0.724 ± 0.149^†^	0.793 ± 0.123^†^	0.624 ± 0.108^†^	0.426 ± 0.187^†^	0.304 ± 0.477	4.4
nnU-Net AMDU (ours)	0.772 ± 0.097	**0.810** **±0.124**	**0.718** **±0.097**	0.262 ± 0.160	**0.197** **±0.228**	**1.6**
MSSEG-*unseen test dataset*						
nnU-Net no DA	0.515 ± 0.198^†^	0.468 ± 0.145^†^	0.628 ± 0.188^†^	0.684 ± 0.227^†^	0.877 ± 2.042^†^	4.2
nnU-Net^*^	0.588 ± 0.169^†^	0.479 ± 0.163^†^	0.619 ± 0.188^†^	**0.281** **±0.197**	0.516 ± 0.668^†^	2.6
nnU-Net^*^ preprocessing^**^	0.548 ± 0.185^†^	0.442 ± 0.168^†^	0.584 ± 0.186^†^	0.337 ± 0.229^†^	0.493 ± 0.330^†^	3.6
Dinsdale et al. (36)	0.564 ± 0.204^†^	0.548 ± 0.150	**0.740** **±0.179**	0.708 ± 0.204^†^	0.828 ± 2.189^†^	3.0
nnU-Net AMDU (ours)	**0.635** **±0.164**	**0.563** **±0.156**	0.700 ± 0.171	0.384 ± 0.199	**0.420** **±0.785**	**1.6**
MSLJ-*unseen test dataset*						
nnU-Net no DA	0.475 ± 0.183^†^	0.357 ± 0.146^†^	0.375 ± 0.108^†^	0.476 ± 0.262^†^	0.519 ± 0.141^†^	4.8
nnU-Net^*^	0.523 ± 0.194^†^	0.403 ± 0.178^†^	0.389 ± 0.120^†^	**0.151** **±0.151**	0.507 ± 0.180^†^	2.4
nnU-Net^*^ preprocessing^**^	0.507 ± 0.181^†^	0.384 ± 0.156^†^	0.431 ± 0.100^†^	0.194 ± 0.183^†^	0.512 ± 0.149^†^	3.0
Dinsdale et al. (36)	0.501 ± 0.171^†^	0.389 ± 0.137^†^	0.401 ± 0.111^†^	0.492 ± 0.264^†^	0.468 ± 0.145^†^	3.4
nnU-Net AMDU (ours)	**0.574** **±0.170**	**0.475** **±0.161**	**0.479** **±0.108**	0.273 ± 0.196	**0.371** **±0.165**	**1.4**
ISBI - *unseen test dataset*						
nnU-Net no DA	0.535 ± 0.149^†^	0.398 ± 0.154^†^	0.500 ± 0.157^†^	0.353 ± 0.178^†^	0.554 ± 0.169^†^	4.6
nnUnet^*^	0.609 ± 0.137^†^	0.477 ± 0.165^†^	0.543 ± 0.131^†^	**0.154 ± 0.098**	0.472 ± 0.190^†^	2.2
nnU-Net preprocessing^**^	0.540 ± 0.139^†^	0.393 ± 0.143^†^	0.574 ± 0.119	0.191 ± 0.115	0.578 ± 0.149^†^	3.4
Dinsdale et al. (36)	0.588 ± 0.130^†^	0.471 ± 0.150^†^	0.522 ± 0.158^†^	0.437 ± 0.146^†^	0.428 ± 0.210^†^	3.4
nnU-Net AMDU (ours)	**0.674** **±0.115**	**0.560** **±0.154**	**0.575** **±0.138**	0.203 ± 0.104	**0.366** **±0.191**	**1.4**

Values are mean ± standard deviation, while bold indicates best results for each performance metric for methods in direct comparison. DA, data augmentation; AMDU, adaptive multi-stage domain unlearning. ^*^Predefined default trainer in nnU-Net. ^**^Preprocessing done on train and test splits with denoise and N4 bias correction. ^†^Mean significantly different (adj. *p* < 0.05; Bonferroni correction) from *nnU-Net AMDU*, per Wilcoxon signed-rank test.

**Figure 5:**
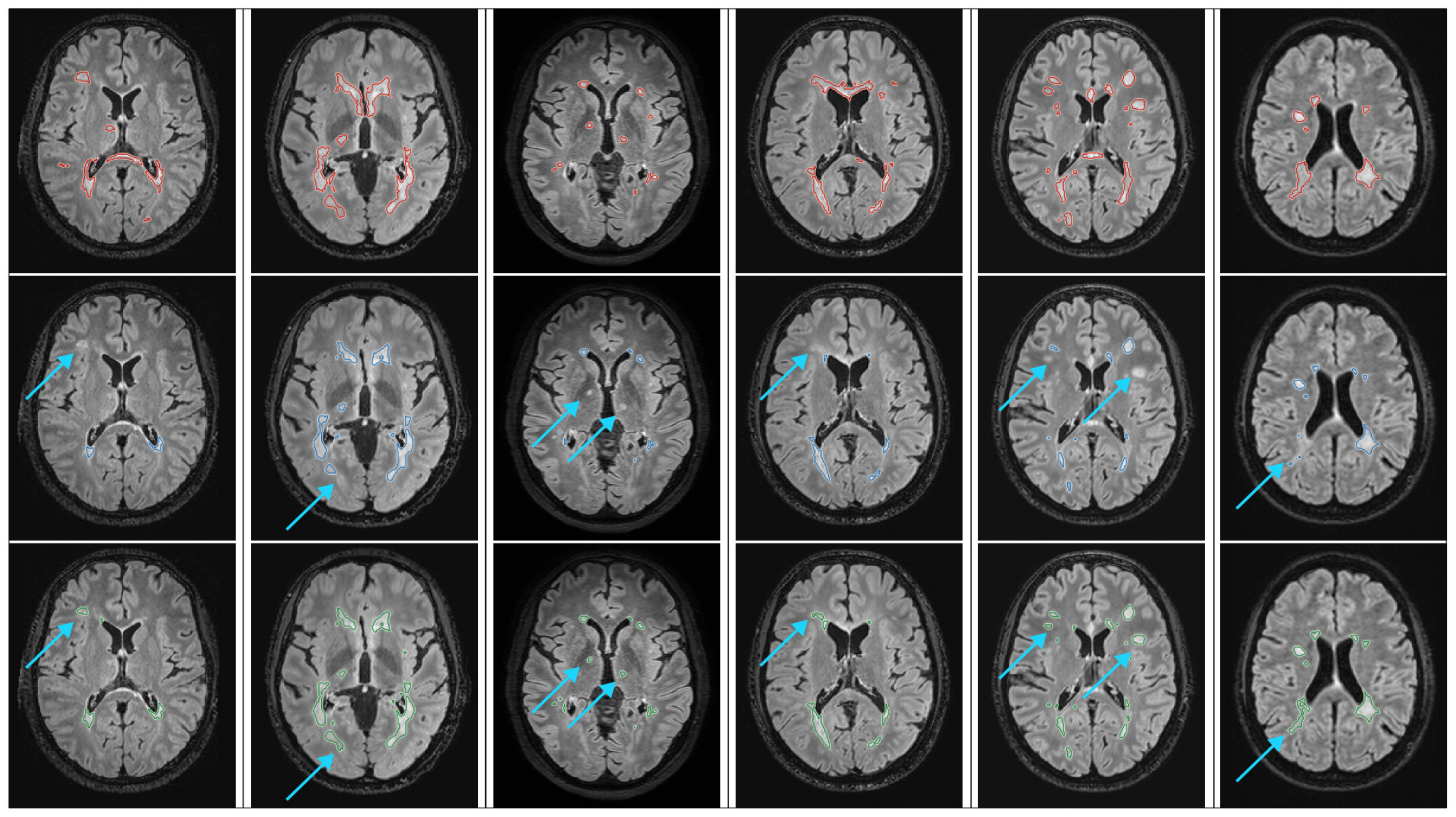
Axial cross-sections of MSSEG 2016 MR scans with superimposed manual **(top row)** and second-best default nnU-Net **(middle row)** and the proposed AMDU model segmentations **(bottom row)**. Arrows highlight important differences.

When applying models to the unseen scanner data in MSSEG, MSLJ, and ISBI datasets, we observe a larger gap between methods, with DSC scores ranging from 0.52–0.64, 0.48–0.57, and 0.54–0.67, respectively. The proposed AMDU outperformed other tested models, with significant increases in DSC, TPR, and LTPR [except in one comparison to Dinsdale et al. (36)] and a significantly lower RVE metric value. Similar to the seen dataset, the LFDR was significantly higher compared to all other model results, but these FPs were small based on small RVE values (Equation 9). Figure 5 visualizes axial MRI cross-sections with superimposed manual, nnU-Net, and AMDU segmentations; the highlights clearly indicate the higher sensitivity to lesions of the AMDU model.

**Figure 6:**
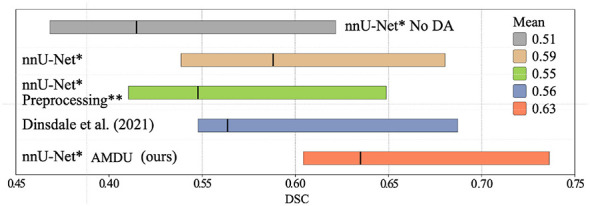
DSC with 95% confidence intervals for MSSEG 2016 unseen test set. For notes (*,**) see caption of Table 4.

**Figure 7:**
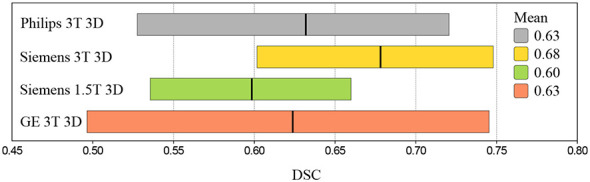
DSC with 95% confidence intervals per source domain of MSSEG unseen test set for nnU-Net AMDU.

The 95% CIs for the DSC values on the MSSEG unseen test set in Figure 6 indicate improved results by the AMDU compared to other tested methods. Furthermore, Figure 7 shows that the proposed strategy had consistent DSC values across different scanners in MSSEG, albeit slightly higher for the Siemens 3T 3D.

### 5.3 State-of-the-art comparison

Methods evaluated on the same public unseen test sets as used in this study, namely MSSEG, MSLJ, and/or ISBI, were considered, enabling a direct and reproducible comparison under shared benchmarking conditions. As summarized in Table 5, the selected methods span diverse model architectures and training datasets differ in their input modalities and represent complementary domain-shift compensation strategies, including implicit robustness through preprocessing and augmentation, test-time adaptation, and train-time adaptation.

**Table 5:** Segmentation evaluation on three public datasets (MSSEG, MSLJ, ISBI; cf. test splits in Table 1).

Model	DSC↑	TPR↑	LTPR↑	LFDR↓	RVE↓
MSSEG - *unseen test dataset*					
nnUNet*	0.537 ± 0.190	0.409 ± 0.188^†^	0.519 ± 0.204^†^	0.365 ± 0.202	0.548 ± 0.217
LST-AI*	0.659 ± 0.159	0.675 ± 0.172^†^	**0.815** **±0.170**^†^	0.452 ± 0.189	0.094 ± 0.568
samseg-7.3.2^*^	0.574 ± 0.177	0.518 ± 0.179	0.369 ± 0.171^†^	0.425 ± 0.205	**0.041** **±1.677**
nnUNet FT 1	0.62 ± 0.06	0.63 ± 0.06^†^	—	—	—
nnUNet FT 3	0.68 ± 0.06	0.67 ± 0.06^†^	—	—	—
nnUNet FT 5	**0.70** **±0.05**	**0.72** **±0.06**^†^	—	—	—
nnU-Net AMDU (ours)	0.635 ± 0.164	0.563 ± 0.15	0.700 ± 0.171	0.384 ± 0.199	0.420 ± 0.785
MSLJ - *unseen test dataset*					
nnUNet^*^	0.443 ± 0.242^†^	0.325 ± 0.200^†^	0.215 ± 0.116^†^	0.367 ± 0.274	0.647 ± 0.216^†^
LST-AI^*^	**0.743** **±0.104**^†^	**0.703** **±0.138**^†^	**0.617** **±0.126**^†^	**0.175** **±0.111**	**0.122** **±0.154**^†^
samseg-7.3.2^*^	0.480 ± 0.250	0.393 ± 0.231	0.192 ± 0.145^†^	0.494 ± 0.255^†^	0.496 ± 0.289
DeepLesionBrain^*^	0.554 ± 0.214	0.476 ± 0.223	0.283 ± 0.102^†^	0.370 ± 0.222	0.337 ± 0.254
nnU-Net AMDU (ours)	0.574 ± 0.170	0.475 ± 0.161	0.479 ± 0.108	0.273 ± 0.196	0.371 ± 0.165
ISBI - *unseen test dataset*					
nnUNet^*^	0.515 ± 0.122^†^	0.381 ± 0.123^†^	0.365 ± 0.144^†^	0.315 ± 0.148^†^	0.545 ± 0.130^†^
LST-AI^*^	0.610 ± 0.126	0.541 ± 0.151	0.552 ± 0.150	0.391 ± 0.132^†^	0.248 ± 0.174^†^
samseg-7.3.2^*^	0.610 ± 0.111	0.556 ± 0.113	0.402 ± 0.140^†^	0.374 ± 0.099^†^	0.167 ± 0.290^†^
nnUNet FT 1	0.63 ± 0.15	0.34 ± 0.19^†^	—	—	—
nnUNet FT 3	0.82 ± 0.02^†^	0.73 ± 0.05^†^	—	—	—
nnUNet FT 5	**0.76** **±0.07**^†^	**0.60** **±0.11**	—	—	—
One-shot DA (61 test)^***^	0.58 ± 0.16^†^	0.48 ± 0.19	—	—	—
DeepLesionBrain^*^	0.607 ± 0.128	0.518 ± 0.165	0.475 ± 0.127^†^	0.343 ± 0.139^†^	0.328 ± 0.209
nnU-Net AMDU (ours)	0.674 ± 0.115	0.560 ± 0.154	0.575 ± 0.138	0.203 ± 0.104	0.366 ± 0.191

Values are mean ± standard deviation, while bold indicates the best results for each performance metric. AMDU, adaptive multi-stage domain unlearning. ^*^Results were sourced from Supplementary Data 1 in Wiltgen et al. (19). ^**^Results were sourced from Table 2 in Coppola et al. (35). ^**^Results were sourced from Table 4 in Valverde et al. (57). ^†^Mean significantly different (*p* < 0.05) from the *nnU-Net AMDU*, as per independent sample *t*-test.

The state-of-the-art comparison in Table 5 indicates that on MSSEG, the proposed AMDU achieved DSC and RVE comparable to those of stronger external methods, while significantly improving sensitivity-related metrics over the default nnU-Net and SAMSEG. On the same dataset, the added test-time adaptation operating points from Coppola et al. (35) provide a clearer supervision-budget perspective: nnUNet FT 1 and FT 3 did not yield a significant DSC advantage over AMDU, although both improved TPR, and FT 5 achieved the strongest overall adapted performance. This suggests that a small amount of target-domain supervision can improve sensitivity before producing a clear overlap advantage, whereas more extensive post-deployment adaptation begins to surpass the source-only regime more consistently. On MSLJ, AMDU significantly outperformed the default nnU-Net in DSC, TPR, LTPR, and RVE, and also improved LTPR relative to SAMSEG and DeepLesionBrain, but remained significantly below LST-AI in DSC, TPR, LTPR, and RVE.

On ISBI, AMDU showed the most favorable overall balance among source-only methods: compared with the default nnU-Net, it achieved significant improvements across all reported metrics, and relative to SAMSEG and DeepLesionBrain, it significantly improved lesion-wise sensitivity and false discovery rate. The low-resource target-adaptive baselines further refine this picture. The One-shot DA model of (57) and nnUNet FT 1 did not outperform AMDU, whereas nnUNet FT 3 and FT 5 from Coppola et al. (35) achieved higher adapted performance, indicating that once several target-domain cases are available, post-deployment adaptation can offer a measurable advantage. Conversely, LST-AI remained statistically comparable to AMDU in DSC, TPR, and LTPR, but achieved significantly lower LFDR and RVE. Overall, these tests suggest that AMDU is most competitive in the strict source-only or very-low-target-supervision regime, whereas multimodal or more strongly target-adapted methods may retain advantages in false-positive control, volume error, or peak overlap once target-domain samples are introduced.

## 6 Discussion

This study introduced and comparatively evaluated a novel ADMU strategy, dubbed AMDU, for WML segmentation, using four established public datasets and evaluation metrics. The strategy was implemented into the state-of-the-art nnU-Net framework and evaluated in a source-only setting on seen and unseen scanner domains. The main implication is that domain robustness in WML segmentation can be improved at training time by reducing scanner-related discriminability in learned features, without requiring target-domain samples, retraining at inference time, or multimodal input.

This interpretation is supported by the ablation evidence (Table 2; Figures 3, 4), the direct strategy comparison (Section 5.2; Table 4), and the external comparison with recent methods (Section 5.3; Tables 5, 6).

**Table 6:** Overview of state-of-the-art lesion segmentation methods.

Model	Architecture	Input modalities	Train set (#patients/#scans)	Domain shift compensation strategy
nnUNet Isensee et al. (43)	3D U-Net	T1w, FLAIR	Private (491/491)	Image and space standardization, intensity augmentation
LST-AI Wiltgen et al. (19)	Ensemble of 3D U-Nets	T1w, FLAIR	Private (491/491)	Image and space standardization, intensity augmentation, ensemble averaging
samseg-7.3.2 Cerri et al. (34)	Generative Bayesian model with deformable atlas + EM	T1w, FLAIR	SAMSEG Atlas + UH Basel dataset (20+54/20+108)	Space standardization and test-time MRI sequence adaptation
DeepBrainLesion Kamraoui et al. (22)	Ensemble of compact 3D CNNs + AssemblyNet	T1w, FLAIR	Private + MSSEG2016train (43+15/43+15)	Distributed regional network voting (akin to ensemble averaging) and low-resolution artifact simulation via augmentation
One-shot DA Valverde et al. (57)	11-layer CNN	T1w, FLAIR	MICCAI2008+MSSEG2016 + 1 *target domain scan* (20/15+*X*)	One-shot test-time adaptation of ultimate three FC layers of pre-trained 11-layer CNN to target domain
nnUNet FT *X* Coppola et al. (35)	3D U-net	T1w, T2w, FLAIR	ICPR2024 + *X* *target domain scans* (90+*X*/114+*X*)	*X*-shot test-time adaptation of pre-trained nnUnet to target domain
nnU-Net AMDU (ours)	3D U-net	FLAIR	WMH2017 (40/40)	Source-only train-time adaptation via domain feature suppression

UH, University Hospital; EM, expectation maximization; CNN, convolutional neural network; ICPR2024-Rondinella et al. (59); WMH2017-Kuijf et al. (24); FC, fully connected; AMDU, adaptive multi-stage domain unlearning.

### 6.1 Adaptive multi-stage domain unlearning

Prior research has explored domain unlearning strategies in medical imaging, notably for brain age regression and tissue segmentation (36) and for T1-weighted scans harmonization (16). While conceptually related, these implementations differ in that the domain classifier is attached solely to the U-Net bottleneck features. Furthermore, Dinsdale et al. (36) injected features from the segmentation output into the domain classifier, thereby unlearning the decoder as well. We experimentally found this to result in unstable training loss dynamics and worse segmentation performance, requiring extensive hyperparameter tuning to achieve a reasonable outcome.

Compared with this literature, the main contribution of AMDU is not the unlearning concept, but a different way of organizing it within the segmentation network. Rather than treating domain suppression as a single bottleneck-level adversarial constraint, AMDU distributes it across multiple encoder depths and couples it to an adaptive schedule. This recasts unlearning as a staged representation-control mechanism that is better aligned with the hierarchical structure of nnU-Net and the need to preserve task-relevant detail.

The stated hypothesis is that the *efficacy of strategies involving modifications or constraints on the latent space depends on the level and/or depth of segmentation model supervision during training* is supported by our findings in Table 3. Models with deeper supervision showed a larger reduction in domain-classifier discriminability, as reflected in accuracies closer to random output (around 50%, cf. Figure 3). This pattern was not observed when unlearning the earlier stages (1, 2, and 3) or all stages (1–6), which aligns with broader representation-learning arguments that nuisance suppression is not uniformly effective across network depth. In the present setting, and within the explored configurations, the deeper encoder stages appear to be the more appropriate targets for reducing scanner-related signal available to the auxiliary classifiers, whereas applying the same to higher-resolution features conflicts with task-relevant detail preservation (cf. Figure 3, settings *A*–*D*).

The second important contribution is the adaptive schedule 3.3.1. Unlike fixed alternation rules commonly used in adversarial or confusion-based training, AMDU delays suppression until a stable domain signal is present. This makes unlearning conditional rather than continuous, aligning with curriculum-style optimization (50) and better suited to the segmentation setting, where premature suppression of nuisances can compete with early task formation. Supporting evidence is provided in Table 3 and Figures 3, 4.

### 6.2 Effectiveness of domain-shift generalization strategies

The comparative evidence in Section 5.2 and Table 4 suggests that the key issue is not simply exposure to more variability, but how that variability is handled during training. Standard augmentation improved robustness, indicating that synthetic variability remains an important baseline defense against domain shift. However, the gains from AMDU imply that variability injection alone is insufficient when scanner-identifying information remains encoded in latent features. The extended external comparison further shows that train-time robustness induction and test-time adaptation operate in different regimes: AMDU improves zero-shot transfer without target data, whereas few-shot target-adaptive methods can leverage even 1–5 target cases to selectively improve cross-domain performance after deployment.

The consistently inferior performance of preprocessing-based harmonization in Table 4 has broader implications for scanner-robust WML segmentation. Intensity manipulation appears to reduce nuisance variability at the cost of removing information useful for lesion delineation. This interpretation aligns with the case-level and cross-dataset trends in Section 5.2 and with earlier reports that achieved good generalization without skull stripping and/or bias field correction in brain and WML segmentation (19, 34) and in tasks such as brain age regression (60).

### 6.3 State-of-the-art comparison

The proposed AMDU is an effective source-only, train-time robustness strategy that preserves zero-shot inference at test time while delivering competitive cross-dataset performance compared with recent alternatives across several families of domain-shift mitigation (Table 6). These include source-free or domain-generalization-oriented methods such as DeepLesionBrain (22), multimodal ensemble-based robustness strategies such as LST-AI (19), generative or harmonization-based methods such as SAMSEG (34), and explicit target-adaptive methods such as one-shot adaptation by Valverde et al. (57) and few-shot adaptation by Coppola et al. (35). These findings suggest that robust cross-domain WML segmentation does not depend on a single recipe but can be achieved through different combinations of architectural capacity, modality richness, aggregation, source-only robustness induction, and target-domain adaptation. Within this landscape, AMDU occupies a complementary position: unlike few-shot or other target-adaptive strategies, it does not require target-domain samples or parameter updates during inference, yet still achieves competitive performance. This supports the view that train-time unlearning can serve as a strong zero-shot baseline.

The added test-time adaptation variants make the trade-off more concrete. On MSSEG, AMDU remained comparable to the 1-shot and 3-shot nnUNet fine-tuning variants in DSC, while the larger 5-shot adaptation setting provided the clearest advantage in adaptation. On ISBI, the single-case target-adaptive baselines, namely One-shot DA and nnUNet FT 1, did not exceed AMDU, whereas nnUNet FT 3 and FT 5 achieved higher adapted overlap and sensitivity. This pattern suggests that the practical breakpoint at which target adaptation becomes clearly advantageous may lie above the strict one-shot regime and may depend on both dataset and architecture. In other words, AMDU is most attractive when no target-domain data are available or when only extremely limited adaptation is feasible, whereas test-time adaptation becomes increasingly compelling as the supervision budget grows from one to several target-domain cases.

Simultaneously, this broader comparison should be interpreted cautiously. Our experiments showed that simple intensity-based preprocessing and harmonization were detrimental to addressing domain shift, whereas more advanced and heterogeneous strategies, including source-free robustness induction, multimodal context, ensembling, minimal-instance or few-shot adaptation, and explicit post-deployment adaptation, may provide complementary advantages. Accordingly, AMDU appears best viewed not as a universal replacement but as a competitive, practical alternative that may also be combined with other mitigation strategies when target data are available.

### 6.4 Annotation variability and benchmarking challenges

Performance differences between seen and unseen sets cannot be attributed solely to domain shift. Table 4 shows a substantial and consistent gap between the seen and unseen subsets, and within the unseen subsets, for each tested model or strategy. Hence, beyond scanner variability, other sources of variability in publicly available datasets affect the consistency and objectivity of benchmarking efforts.

**Figure 8:**
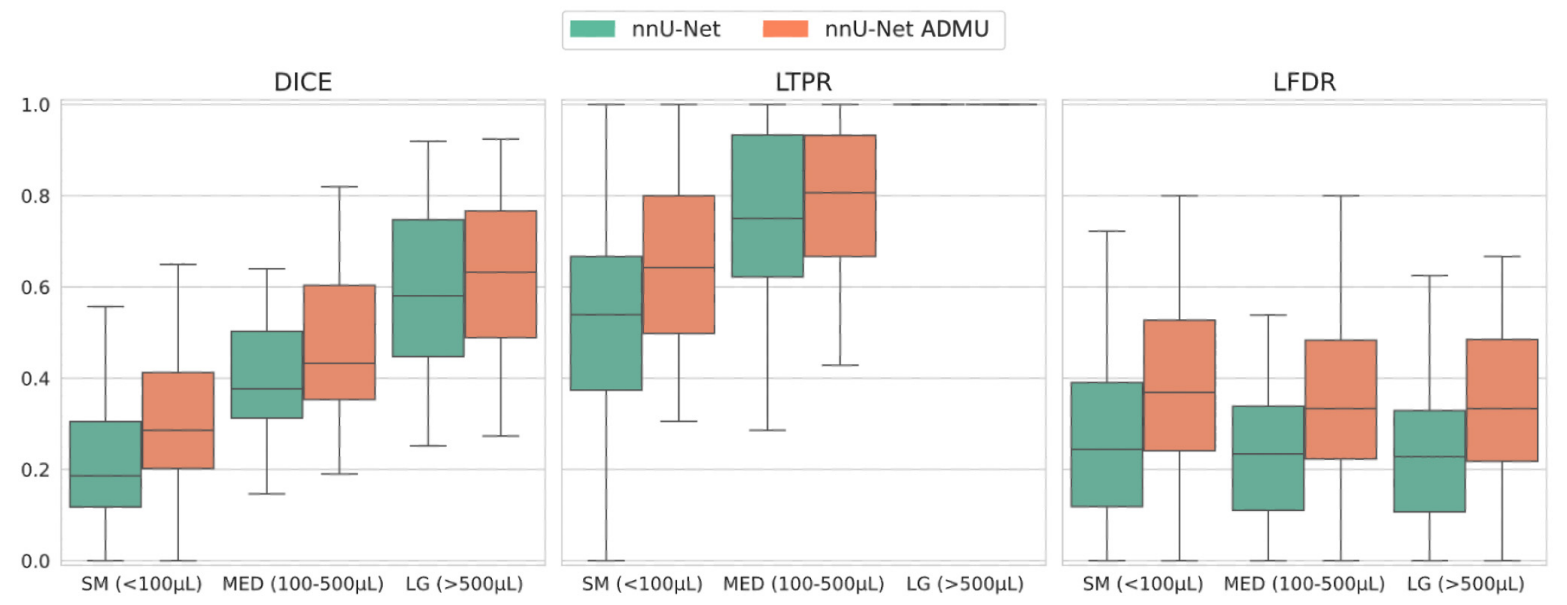
Lesion segmentation performance according to lesion size, i.e., small (SM), medium (MED), and large (LG); obtained on the MSSEG dataset.

Lesion size affects segmentation performance and its assessment. Figure 8 shows that lesion size substantially affected the baseline nnU-Net and the AMDU models: smaller lesions had lower segmentation overlap, subject to quantization effects, and lower lesion-wise detection rates, depending on the chosen detection thresholds. Across all lesion-size groups, however, the proposed AMDU model consistently achieved higher DSC and LTPR than the baseline nnU-Net, indicating improved sensitivity, particularly for small lesions (volume < 100 μl), for which the median LTPR increased from 0.55 to 0.67. Although LFDR was also consistently higher for AMDU across lesion-size groups (median 0.38 vs. 0.25), this was accompanied by improved overlap and lesion detection performance, suggesting AMDU provides a more favorable overall balance between sensitivity and precision.

**Figure 9:**
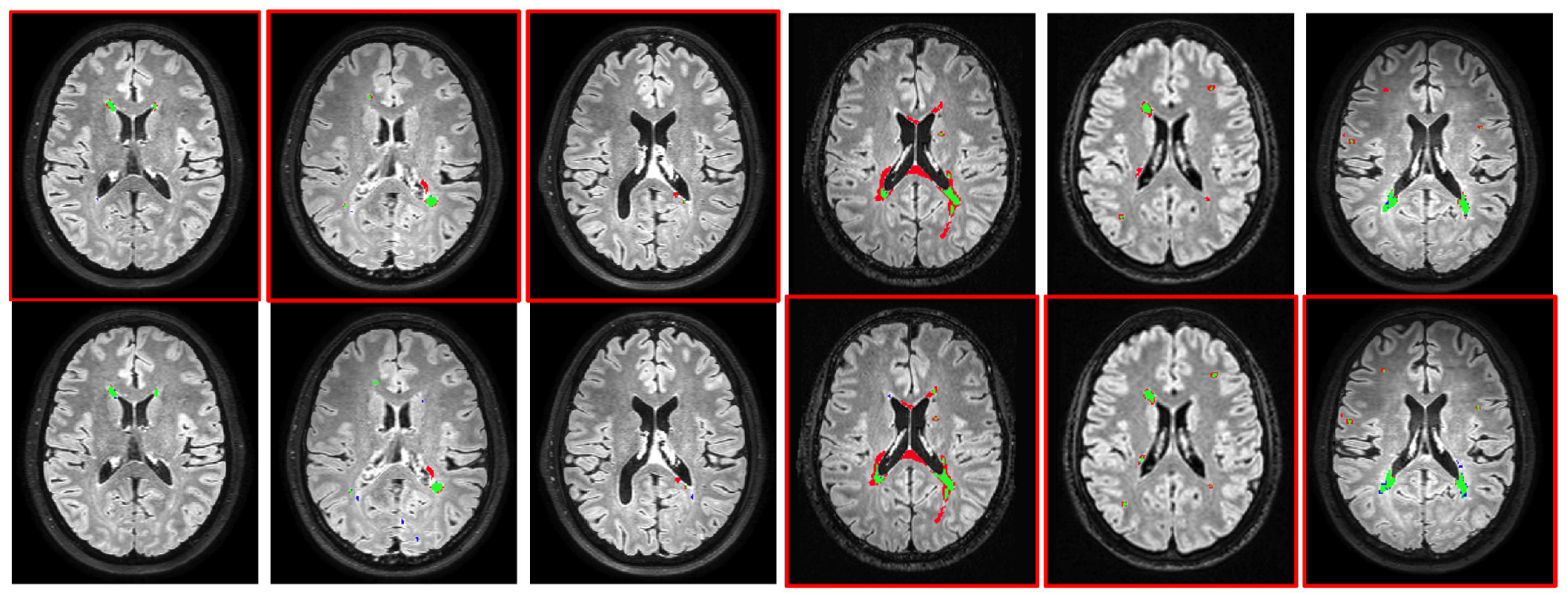
Axial cross-sections of six MSSEG 2016 MR scans (columns) for the nnU-Net baseline **(top row)** and the proposed nnU-Net AMDU **(bottom row)** with superimposed true positives (green), false positives (blue) and false negatives (red) **(bottom row)**. Red frame indicates the best result according to the DSC metric. Overall, the nnU-Net baseline was best in 5, while the proposed nnU-Net AMDU was best in 47 out of 52 test cases.

Inconsistencies in ground truth WML segmentations can confound performance comparisons and hinder the development of universally robust models. The four public datasets used in this study involved different numbers of expert raters who annotated lesions using different tools and protocols, inevitably introducing inconsistencies into the ground truth or reference WML segmentations. Compared to nnU-Net, the proposed AMDU model achieved significantly higher DSC, TPR, and LTPR, and lower RVE on the unseen test set, while exhibiting a higher LFDR. At the case level, our analysis of the 52 MSSEG 2016 unseen test scans shows that nnU-Net was superior in only five cases, whereas AMDU performed best in 47 cases. This pattern informs the interpretation of the false-positive burden. As illustrated in Figure 9, AMDU typically produces more false-positive voxels (cf. columns 2, 3, 4, and 6), yet visual inspection suggests that many of these may correspond to plausible lesions that were not annotated in the reference standard. In subjects with very few lesions, such false positives can disproportionately affect LFDR, shifting the balance in favor of the baseline. Nonetheless, the lower RVE from AMDU indicates that the false positives tend to be smaller. Independent qualitative clinical validation would be needed to determine how many apparent false positives actually correspond to subtle lesions missing from the reference standard.

### 6.5 Mitigation of study limitations

Segmentation of WMLs is an active and competitive research field, with many effective methods available beyond those evaluated in this study. To enable future comparative analyses, we trained and evaluated our models on four publicly available datasets (Table 1). Furthermore, we integrated the proposed AMDU strategy into the nnU-Net framework, which has consistently delivered state-of-the-art results across a wide range of medical segmentation challenges (43). The source code is publicly available,^3^ enabling reproducibility, extension, and fair comparison by the broader research community.

One limitation of this study is that the proposed approach was evaluated exclusively on the WML segmentation task. Because the proposal is architectural rather than lesion-specific, it appears applicable to other segmentation problems affected by site, scanner, or protocol variability. While beyond the scope of this study, the seamless integration of AMDU into the nnU-Net framework, along with its adaptive training schedule, minimal hyperparameter tuning, and publicly available source code makes it readily applicable to other datasets and segmentation tasks. This flexibility encourages further exploration and validation of the method in broader clinical and research settings.

Another limitation is the relatively small size of the training dataset of 40 cases. This choice was intentional, as we prioritized the use of publicly available datasets to ensure reproducibility and to provide a transparent foundation that others can build upon. While this approach supports openness and comparability, it inherently restricts the biological and scanner variability available during training, which may impact segmentation performance and generalization capability. Notably, the LST-AI model (19) was trained on a substantially larger in-house acquired and annotated dataset of 491 cases and evaluated on the same unseen datasets, reporting similar performance on MSSEG (all cases), superior on MSLJ and inferior on ISBI.^4^ Besides a larger train set, which may scale the performance, their model inputs both T1-weighted and FLAIR scans, whereas AMDU used only FLAIR. Interestingly, the other methods tested in Wiltgen et al. (19) all showed inferior performance compared to AMDU on all three unseen test sets.

These findings indicate that multimodal inputs can provide complementary information, yet they also show that a carefully designed FLAIR-only model—trained on heterogeneous public datasets and equipped with ADMU—can achieve performance competitive with a substantially larger multimodal system, and in some cases surpass it on unseen scanners. Moreover, the WMH dataset forming our training set contains vascular-origin lesions that differ in phenotype from MS lesions, likely increasing task difficulty on unseen MS lesions, but also limiting potential gains from additional modalities.

Most models use multimodal MRI for WML segmentation, typically T1-weighted and FLAIR (19, 22), or even more (34, 35). We limited our experiments to using a single FLAIR modality for WML segmentation as this offers several practical and methodological advantages. FLAIR imaging provides high sensitivity and contrast for detecting WMLs, especially in periventricular and juxtacortical regions, making it well-suited as a standalone modality. Relying on one modality simplifies preprocessing pipelines, reduces inter-modality registration errors, and eliminates the need for inter-modality harmonization, which can introduce variability. Clinically, single-modality approaches increase applicability, particularly in retrospective studies or settings where only FLAIR is routinely acquired. Moreover, single-modality models are significantly less memory- and computation-intensive, enabling training and inference on a wider range of hardware, including resource-constrained environments. This increases the method's accessibility and usability in both research and clinical settings.

Although the proposed ADMU framework is compatible with other forms of nuisance-variable suppression (e.g., modality, age, or sex unlearning), extending it to other anatomies or multi-modal inputs would require additional architectural considerations, such as modality-specific encoders or separate unlearning branches, and therefore represent an important direction for future research.

### 6.6 Conclusion

The proposed AMDU enhancement of the nnU-Net segmentation method shows strong potential for clinical application in estimating lesion-based biomarkers, such as lesion volume and count, which are critical for diagnosis and monitoring treatment response (4). It also shows promise for cortical lesion segmentation (61) and for predicting MS progression (5). The nnU-Net AMDU achieved low RVE, directly reflecting good accuracy and low uncertainty in lesion volume estimation. Additionally, its performance on lesion-wise metrics, particularly the high LTPR and balanced LFDR, indicates reliable lesion count estimation, with high LTPR being especially important for detecting new or enlarging lesions in longitudinal studies. Therefore, we conclude that nnU-Net AMDU reasonably addresses the clinical demands for an accurate, versatile, and robust biomarker extraction method in MS imaging.

## Data Availability

The original contributions presented in the study are included in the article/supplementary material, further inquiries can be directed to the corresponding author.

## Footnotes

1 nnU-Net: https://github.com/MIC-DKFZ/nnUNet.

2 AntsPy: https://github.com/ANTsX/ANTsPy.

3 Source code is available at https://github.com/Pubec/nnunetv2-unlearning.

4 Cf. Table 4, based on Supplementary Data 1 in Wiltgen et al. (19).

## References

[1] Kaur A Kaur L Singh A. State-of-the-art segmentation techniques and future directions for multiple sclerosis brain lesions. Arch Comput Methods Eng. (2020) 28:1–27. doi: 10.1007/s11831-020-09403-7

[2] Eshaghi A Young AL Wijeratne PA Prados F Arnold DL Narayanan S . Identifying multiple sclerosis subtypes using unsupervised machine learning and MRI data. Nat Commun. (2021) 12:2078. doi: 10.1038/s41467-021-22265-2 33824310 PMC8024377

[3] McGinley MP Goldschmidt CH Rae-Grant AD. Diagnosis and treatment of multiple sclerosis: a review. JAMA (2021) 325:765–79. doi: 10.1001/jama.2020.26858 33620411

[4] Popescu V Agosta F Hulst HE Sluimer IC Knol DL Sormani MP . Brain atrophy and lesion load predict long term disability in multiple sclerosis. J Neurol Neurosurg Psychiatry (2013) 84:1082–91. doi: 10.1136/jnnp-2012-304094 23524331

[5] Oship D Jakimovski D Bergsland N Horakova D Uher T Vaneckova M . Assessment of T2 lesion-based disease activity volume outcomes in predicting disease progression in multiple sclerosis over 10 years. Mult Scler Relat Disord. (2022) 67:104187. doi: 10.1016/j.msard.2022.104187 36150263

[6] Sastre-Garriga J Pareto D Battaglini M Rocca MA Ciccarelli O Enzinger C . MAGNIMS consensus recommendations on the use of brain and spinal cord atrophy measures in clinical practice. Nat Rev Neurol. (2020) 16:171–82. doi: 10.1038/s41582-020-0314-x 32094485 PMC7054210

[7] Alzubaidi L Zhang J Humaidi AJ Al-Dujaili A Duan Y Al-Shamma O . Review of deep learning: concepts, CNN architectures, challenges, applications, future directions. J Big Data (2021) 8:53. doi: 10.1186/s40537-021-00444-8 33816053 PMC8010506

[8] D'Amour A Heller K Moldovan D Adlam B Alipanahi B Beutel A . Underspecification presents challenges for credibility in modern machine learning. J Mach Learn Res. (2022) 23:1–61. doi: 10.5555/3586589.3586815

[9] Commowick O Istace A Kain M Laurent B Leray F Simon M . Objective evaluation of multiple sclerosis lesion segmentation using a data management and processing infrastructure. Sci Rep. (2018) 8:13650. doi: 10.1038/s41598-018-31911-7 30209345 PMC6135867

[10] Masson A Combès B Ardellier FD Rabaste S Cloarec P Wdowik Q . Performances of experts and automated methods on new multiple sclerosis lesions detection: insights from the MSSeg2 challenge. Sci Rep. (2026) 16:9233. doi: 10.1038/s41598-026-52150-1 42168373 PMC13402319

[11] Isensee F Jaeger PF Kohl SA Petersen J Maier-Hein KH. nnU-Net: a self-configuring method for deep learning-based biomedical image segmentation. Nat Methods (2021) 18:203–11. doi: 10.1038/s41592-020-01008-z 33288961

[12] Guan H Liu M. Domain adaptation for medical image analysis: a survey. IEEE Trans Biomed Eng. (2022) 69:1173–85. doi: 10.1109/TBME.2021.3117407 34606445 PMC9011180

[13] Zhang L Wang X Yang D Sanford T Harmon S Turkbey B . Generalizing deep learning for medical image segmentation to unseen domains via deep stacked transformation. IEEE Trans Med Imaging (2020) 39:2531–40. doi: 10.1109/TMI.2020.2973595 32070947 PMC7393676

[14] Basaran BD Matthews PM Bai W. New lesion segmentation for multiple sclerosis brain images with imaging and lesion-aware augmentation. Front Neurosci. (2022) 16:1–14. doi: 10.3389/fnins.2022.1007453 36340756 PMC9633994

[15] Basaran BD Zhang X Matthews PM Bai W. SegHeD: Segmentation of Heterogeneous Data for Multiple Sclerosis Lesions with Anatomical Constraints. (2024). Available online at: https://arxiv.org/abs/2410.01766 (Accessed July 24, 2026).

[16] Cackowski S Barbier EL Dojat M Christen T. ImUnity: a generalizable VAE-GAN solution for multicenter MR image harmonization. Med Image Anal. (2023) 88:102799. doi: 10.1016/j.media.2023.102799 37245434

[17] Hitziger S Ling WX Fritz T D'Albis T Lemke A Grilo J. Triplanar U-Net with lesion-wise voting for the segmentation of new lesions on longitudinal MRI studies. Front Neurosci. (2022) 16:1–13. doi: 10.3389/fnins.2022.964250 36033604 PMC9412001

[18] Katona M Bozsik B Bodnár P Kocsis K Tóth E Szabó N . DenseLes: slice-wise dense network for multiple sclerosis lesion segmentation and classification. Front Neurol. (2026) 17:1–11. doi: 10.3389/fneur.2026.1704317 PMC12979175 41835080

[19] Wiltgen T McGinnis J Schlaeger S Kofler F Voon C Berthele A . LST-AI: a deep learning ensemble for accurate MS lesion segmentation. NeuroImage Clin. (2024) 42:103611. doi: 10.1016/j.nicl.2024.103611 38703470 PMC11088188

[20] Ronneberger O Fischer P Brox T. U-Net: convolutional networks for biomedical image segmentation. In:Navab N Hornegger J Wells WM Frangi AF, editors. Medical Image Computing and Computer-Assisted Intervention – MICCAI 2015. Cham: Springer International Publishing (2015). p. 234–41.

[21] Yang Z Xu P Yang Y Bao BK. A densely connected network based on U-Net for medical image segmentation. ACM Trans Multimedia Comput Commun Appl. (2021) 17:1–14. doi: 10.1145/3446618

[22] Kamraoui RA Ta VT Tourdias T Mansencal B Manjon JV Coupé P. DeepLesionBrain: towards a broader deep-learning generalization for multiple sclerosis lesion segmentation. Med Image Anal. (2022) 76:102312. doi: 10.1016/j.media.2021.102312 34894571

[23] Akkus Z Galimzianova A Hoogi A Rubin DL Erickson BJ. Deep learning for brain MRI segmentation: state of the art and future directions. J Digit Imaging (2017) 30:449–59. doi: 10.1007/s10278-017-9983-4 28577131 PMC5537095

[24] Kuijf H Biesbroek M De Bresser J Heinen R Chen C Van Der Flier W . Data of theWhiteMatter Hyperintensity (WMH) Segmentation Challenge. DataverseNL V1 (2022). Available online at: https://dataverse.nl/dataset.xhtml?persistentId=doi:10.34894/AECRSD

[25] Commowick O Kain M Casey R Ameli R Ferré JC Kerbrat A . Multiple sclerosis lesions segmentation from multiple experts: the MICCAI 2016 challenge dataset. Neuroimage (2021) 244:118589. doi: 10.1016/j.neuroimage.2021.118589 34563682

[26] Hindawi S Szubstarski B Boernert E Tackenberg B Wuerfel J. Federated learning for lesion segmentation in multiple sclerosis: a real-world multi-center feasibility study. Front Neurol. (2025) 16:1–9. doi: 10.3389/fneur.2025.1620469 41001196 PMC12457141

[27] Fortin JP Sweeney EM Muschelli J Crainiceanu CM Shinohara RT Alzheimer's Disease Neuroimaging Initiative. Removing inter-subject technical variability in magnetic resonance imaging studies. NeuroImage (2016) 132:198–212. doi: 10.1016/j.neuroimage.2016.02.036 26923370 PMC5540379

[28] Fortin JP Parker D Tunç B Watanabe T Elliott MA Ruparel K . Harmonization of multi-site diffusion tensor imaging data. Neuroimage (2017) 161:149–70. doi: 10.1016/j.neuroimage.2017.08.047 28826946 PMC5736019

[29] Pomponio R Erus G Habes M Doshi J Srinivasan D Mamourian E . Harmonization of large MRI datasets for the analysis of brain imaging patterns throughout the lifespan. Neuroimage (2020) 208:116450. doi: 10.1016/j.neuroimage.2019.116450 31821869 PMC6980790

[30] Beer JC Tustison NJ Cook PA Davatzikos C Sheline YI Shinohara RT . Longitudinal ComBat: a method for harmonizing longitudinal multi-scanner imaging data. Neuroimage (2020) 220:117129. doi: 10.1016/j.neuroimage.2020.117129 32640273 PMC7605103

[31] Liu S Yap PT. Learning multi-site harmonization of magnetic resonance images without traveling human phantoms. Commun Eng. (2024) 3:1–10. doi: 10.1038/s44172-023-00140-w 38420332 PMC10898625

[32] Kuang S Woodruff HC Granzier R Nijnatten TJAv Lobbes MBI Smidt ML . MSCDA: multi-level semantic-guided contrast improves unsupervised domain adaptation for breast MRI segmentation in small datasets. Neural Netw. 165:119–34. (2023). doi: 10.1016/j.neunet.2023.05.014 37285729

[33] Billot B Greve DN Puonti O Thielscher A Van Leemput K Fischl B . SynthSeg: segmentation of brain MRI scans of any contrast and resolution without retraining. Med Image Anal. (2023) 86:102789. doi: 10.1016/j.media.2023.102789 36857946 PMC10154424

[34] Cerri S Puonti O Meier DS Wuerfel J Mühlau M Siebner HR . A contrast-adaptive method for simultaneous whole-brain and lesion segmentation in multiple sclerosis. Neuroimage (2021) 225:117471. doi: 10.1016/j.neuroimage.2020.117471 33099007 PMC7856304

[35] Coppola E Savardi M Signoroni A. Towards robust and reliable multi-modal 3D segmentation of multiple sclerosis lesions. Pattern Recognit Lett. (2026) 200:115–22. doi: 10.1016/j.patrec.2025.12.008

[36] Dinsdale NK Jenkinson M Namburete AIL. Deep learning-based unlearning of dataset bias for MRI harmonisation and confound removal. Neuroimage (2021) 228:117689. doi: 10.1016/j.neuroimage.2020.117689 33385551 PMC7903160

[37] Guan H Liu Y Yang E Yap PT Shen D Liu M. Multi-site MRI harmonization via attention-guided deep domain adaptation for brain disorder identification. Med Image Anal. (2021) 71:102076. doi: 10.1016/j.media.2021.102076 33930828 PMC8184627

[38] Wolleb J Sandkühler R Bieder F Barakovic M Hadjikhani N Papadopoulou A . Learn to ignore: domain adaptation for multi-site MRI analysis. In:Wang L Dou Q Fletcher PT Speidel S Li S, editors. Medical Image Computing and Computer Assisted Intervention-MICCAI 2022. Cham: Springer Nature Switzerland (2022). p. 725–35.

[39] Bi Y Jiang Z Clarenbach R Ghotbi R Karlas A Navab N. MI-SegNet: Mutual Information-Based US Segmentation forUnseen Domain Generalization. In:Greenspan H Madabhushi A Mousavi P Salcudean S Duncan J Syeda-Mahmood T et al., editors. Medical Image Computing and Computer Assisted Intervention - MICCAI 2023. MICCAI 2023. Lecture Notes in Computer Science, Vol. 14223 (2023). Cham: Springer. doi: 10.1007/978-3-031-43901-8_13

[40] He Y Nath V Yang D Tang Y Myronenko A Xu D. SwinUNETR-V2: stronger swin transformers with stagewise convolutions for 3D medical image segmentation. In:Greenspan H Madabhushi A Mousavi P Salcudean S Duncan J Syeda-Mahmood T et al., editors. Medical Image Computing and Computer Assisted Intervention-MICCAI 2023. Cham: Springer Nature Switzerland (2023). p. 416–26.

[41] Jain S Rajpal N Soni PK. MS-DASPNet: multiple sclerosis lesion segmentation from brain MRI using dual attention and spatial pyramid pooling with transfer learning. Front Comput Neurosci. (2026) 19:1–22. doi: 10.3389/fncom.2025.1713766 41694140 PMC12894251

[42] Alshehri F Muhammad G. Ischemic stroke segmentation by transformer and convolutional neural network using few-shot learning. ACM Trans Multimedia Comput Commun Appl. (2024) 20:1–21. doi: 10.1145/3699513

[43] Isensee F Wald T Ulrich C Baumgartner M Roy S Maier-Hein K . nnU-Net revisited: a call for rigorous validation in 3D medical image segmentation. In:Linguraru MG Dou Q Feragen A Giannarou S Glocker B Lekadir K et al., editors. Medical Image Computing and Computer Assisted Intervention-MICCAI 2024. Cham: Springer Nature Switzerland (2024). p. 488–98.

[44] Christodoulou E Reinke A Houhou R Kalinowski P Erkan S Sudre CH . Confidence intervals uncovered: are we ready for real-world medical imaging AI? In:Linguraru MG Dou Q Feragen A Giannarou S Glocker B Lekadir K , editors. Medical Image Computing and Computer Assisted Intervention-MICCAI 2024. Cham: Springer Nature Switzerland (2024). p. 124–32.

[45] Milletari F Navab N Ahmadi SA. V-Net: fully convolutional neural networks for volumetricmedical image segmentation. In: 2016 fourth international conference on 3D vision (3DV). Stanford, CA: IEEE (2016). p. 565–71. doi: 10.1109/3DV.2016.79

[46] Kline DM Berardi VL. Revisiting squared-error and cross-entropy functions for training neural network classifiers. Neural Comput Appl. (2005) 14:310–8. doi: 10.1007/s00521-005-0467-y

[47] Ganin Y Ustinova E Ajakan H Germain P Larochelle H Laviolette F . Domain-adversarial training of neural networks. J Mach Learn Res. (2016) 17:2096–2030. doi: 10.5555/2946645.2946704

[48] Tzeng E Hoffman J Zhang N Saenko K Darrell T. Deep domain confusion: maximizing for domain invariance. arXiv [preprint]. (2014). arXiv:1412.3474.

[49] Tishby N Zaslavsky N. Deep learning and the information bottleneck principle. In: 2015 IEEE information theory workshop (ITW). (2015). p. 1–5. Available online at: https://ieeexplore.ieee.org/document/7133169

[50] Soviany P Ionescu RT Rota P Sebe N. Curriculum learning: a survey. Int J Comput Vis. (2022) 130:1526–65. doi: 10.1007/s11263-022-01611-x

[51] Lesjak Ž Galimzianova A Koren A Lukin M Pernuš F Likar B . A novel public MR image dataset of multiple sclerosis patients with lesion segmentations based on multi-rater consensus. Neuroinformatics (2018) 16:51–63. doi: 10.1007/s12021-017-9348-7 29103086

[52] Carass A Roy S Jog A Cuzzocreo JL Magrath E Gherman A . Longitudinal multiple sclerosis lesion segmentation: resource and challenge. Neuroimage (2017) 148:77–102. doi: 10.1016/j.neuroimage.2016.12.064 28087490 PMC5344762

[53] Fonov V Evans A McKinstry R Almli C Collins D. Unbiased nonlinear average age-appropriate brain templates from birth to adulthood. Neuroimage (2009) 47:S102. doi: 10.1016/S1053-8119(09)70884-5

[54] Tustison NJ Avants BB Cook PA Zheng Y Egan A Yushkevich PA . N4ITK: improved N3 bias correction. IEEE Trans Med Imaging (2010) 29:1310–20. doi: 10.1109/TMI.2010.2046908 20378467 PMC3071855

[55] Manjón JV Coupé P Martí-Bonmatí L Collins DL Robles M. Adaptive non-local means denoising of MR images with spatially varying noise levels. J Magn Reson Imaging (2010) 31:192–203. doi: 10.1002/jmri.22003 20027588

[56] Tustison NJ Cook PA Holbrook AJ Johnson HJ Muschelli J Devenyi GA . The ANTsX ecosystem for quantitative biological and medical imaging. Sci Rep. (2021) 11:9068. doi: 10.1038/s41598-021-87564-6 33907199 PMC8079440

[57] Valverde S Salem M Cabezas M Pareto D Vilanova JC Ramió-Torrentà L . One-shot domain adaptation in multiple sclerosis lesion segmentation using convolutional neural networks. NeuroImage Clin. (2019) 21:101638. doi: 10.1016/j.nicl.2018.101638 30555005 PMC6413299

[58] Wilcoxon F. Individual comparisons by ranking methods. Biometrics Bulletin (1945) 1:80–3. doi: 10.2307/3001968 18903631

[59] Rondinella A Guarnera F Crispino E Russo G Di Lorenzo C Maimone D . ICPR 2024 competition on multiple sclerosis lesion segmentation–methods and results. In:Antonacopoulos A Chaudhuri S Chellappa R Liu CL Bhattacharya S Pal U, editors. Pattern Recognition. Competitions. Cham: Springer Nature Switzerland (2025). p. 1–16.

[60] Dartora C Marseglia A Mårtensson G Rukh G Dang J Muehlboeck JS . A deep learning model for brain age prediction using minimally preprocessed T1w images as input. Front Aging Neurosci. (2024) 15:1–17. doi: 10.3389/fnagi.2023.1303036 38259636 PMC10800627

[61] Freeman HJ Atalay AS Li J Sobczak E Gilmore N Snider SB . Unsupervised semi-automated MRI segmentation detects cortical lesion expansion in chronic traumatic brain injury. Front Neurol. (2025) 16:1–15. doi: 10.3389/fneur.2025.1640514 41170325 PMC12568329

